# Mesodermal Gene Expression in the Acoel *Isodiametra pulchra* Indicates a Low Number of Mesodermal Cell Types and the Endomesodermal Origin of the Gonads

**DOI:** 10.1371/journal.pone.0055499

**Published:** 2013-02-06

**Authors:** Marta Chiodin, Aina Børve, Eugene Berezikov, Peter Ladurner, Pedro Martinez, Andreas Hejnol

**Affiliations:** 1 Departament de Genètica, Universitat de Barcelona, Barcelona, Spain; 2 Sars International Centre for Marine Molecular Biology, University of Bergen, Bergen, Norway; 3 Institute of Zoology and Center for Molecular Biosciences, University of Innsbruck, Innsbruck, Austria; 4 Hubrecht Institute, Utrecht, The Netherlands; 5 Institució Catalana de Recerca i Estudis Avançats (ICREA), Barcelona, Spain; Ecole Normale Supérieure de Lyon, France

## Abstract

Acoelomorphs are bilaterally symmetric small marine worms that lack a coelom and possess a digestive system with a single opening. Two alternative phylogenetic positions of this group within the animal tree are currently debated. In one view, Acoelomorpha is the sister group to all remaining Bilateria and as such, is a morphologically simple stepping stone in bilaterian evolution. In the other, the group is a lineage within the Deuterostomia, and therefore, has derived a simple morphology from a more complex ancestor. Acoels and the closely related Nemertodermatida and Xenoturbellida, which together form the Acoelomorpha, possess a very limited number of cell types. To further investigate the diversity and origin of mesodermal cell types we describe the expression pattern of 12 orthologs of bilaterian mesodermal markers including *Six1/2*, *Twist, FoxC*, *GATA4/5/6*, in the acoel *Isodiametra pulchra*. All the genes are expressed in stem cells (neoblasts), gonads, and at least subsets of the acoel musculature. Most are expressed in endomesodermal compartments of *I. pulchra* developing embryos similar to what has been described in cnidarians. Our molecular evidence indicates a very limited number of mesodermal cell types and suggests an endomesodermal origin of the gonads and the stem cell system. We discuss our results in light of the two prevailing phylogenetic positions of Acoelomorpha.

## Introduction

The mesoderm is the embryonic germ layer that develops between the endoderm and the ectoderm. It is regarded as a key innovation that led to the diversification of organ systems and cell types present in bilaterally symmetrical animals (Bilateria) [1], [2], [3], [4], [5]. In the Bilateria the mesoderm gives rise to structures such as body wall musculature, supporting skeletons and secondary body cavities (coeloms). In some lineages these body cavities evolved into new organ systems such as the excretory and circulatory system that in turn allowed the evolution of larger body sizes [6], [7], [8]. Thus, the origin and evolution of the mesoderm have been central to formulating hypotheses of the transition from a relatively simple radially symmetric ancestor to a complex bilaterian. A crucial topic in the different scenarios is the homology of coelomic cavities and how often they originated in animals [3], [9], [10], [11]. According to the ‘archicoelomate hypothesis’ or ‘enterocoely hypothesis’ [3], [12] the coelomic cavities of bilaterians evolved from evaginations of the gastric epithelium of a cnidarian polyp-like ancestor. This mode of coelom development (enterocoely) is observed in some extant deuterostomes such as echinoderms and some hemichordate lineages, in which it gives rise to a tripartite organization of coelomic cavities. According to Remane [12], such tripartite organization of body cavities is the ancestral bilaterian state and the acoelomate and “pseudocoelomate” conditions would have arisen by independent reductions of the coeloms in multiple animal lineages [13].

The mesoderm of extant coelomate animals consists of defined muscular layers and coeloms. Coeloms can be lined by a simple epithelium (pleura) or by an epithelio-muscular lining (myo-epithelium), and often both linings are present in the same taxon. A myo-epithelium consists of alternating epithelial cells and epithelio-muscular cells, which are epithelial cells with basally accumulated contractile filaments (mainly actin and myosin). It is supposed that myo-epithelium represent the ancestral contractile cells types [8], [14]. According to some authors, a separation of the contractile myoepithelial cells and the epithelial cells would have occurred in the myo-epithelial lined coelom of the bilaterian last common ancestor (archicoelomate) [15].

A different scenario for the origin of the mesoderm is suggested by the acoeloid-planuloid hypothesis [4], [16], [17]. Here, the separation between the muscular contractile basal portion and epithelial apical portion would have occurred in the endoderm of a planula-like ancestor. In this scenario, individual myocytes, most likely arranged in an orthogonal grid of circular and longitudinal muscles, would be the first type of mesodermal tissue. According to this theory, the last common bilaterian ancestor was an organism that was similarly organized to extant acoelomorphs [18], which possess this type of muscular arrangement. In cnidarians, the sister group of the Bilateria, bilaterian ‘mesodermal’ genes are expressed in the endoderm [2] suggesting that the mesoderm evolved from the endoderm. However, it is an open question as to how and when this transition occurred.

In this study we present the expression patterns of 12 bilaterian mesodermal markers (Fig. S1) in *Isodiametra pulchra* (Acoela, Acoelomorpha). Acoelomorphs are unsegmented, acoelomate worms, sometimes referred as the proxy of the ancestral bilaterian in planuloid-acoeloid theory [4], [16], [19]. Recent molecular phylogenies and most modern phylogenomic approaches have supported this proposition by showing that acoelomorphs branched off the bilaterian tree before the protostome-deuterostome split [20], [21]. However, a different phylogenomic study that applied a site-heterogeneous model shows acoelomorphs as the sister group of the Ambulacraria [22], thereby implying that the morphological simplicity of the acoelomorphs is due to a loss of many characters (*e.g.*, the anus, coelomic cavities and excretory system) possibly by neoteny (paedomorphosis) [23]. Since the phylogenetic position is still in debate [24], we discuss our results in the light of both hypotheses.

The musculature is the most prominent mesodermal derivative in *I. pulchra* and its ontogeny and architecture have been thoroughly studied [18], [25]. Furthermore a mesenchymal tissue, called the parenchyma, fills the body space between the digestive syncytium and the body wall. The parenchyma develops from endomesodermal precursors and it is declared as mesodermal tissue only on the basis of its location in adult worm [26], [27]. Gonads and neoblasts are also located in the parenchyma, but the embryonic origin of these tissues remains unclear [28], [29].

In this study, we compare the expression patterns of mesodermal genes in I. pulchra with the expression of the orthologs in the Bilateria and Cnidaria and try to infer the ancestral condition of bilaterian mesoderm.

## Results

### Anatomy of *I. pulchra*

**Figure 1:**
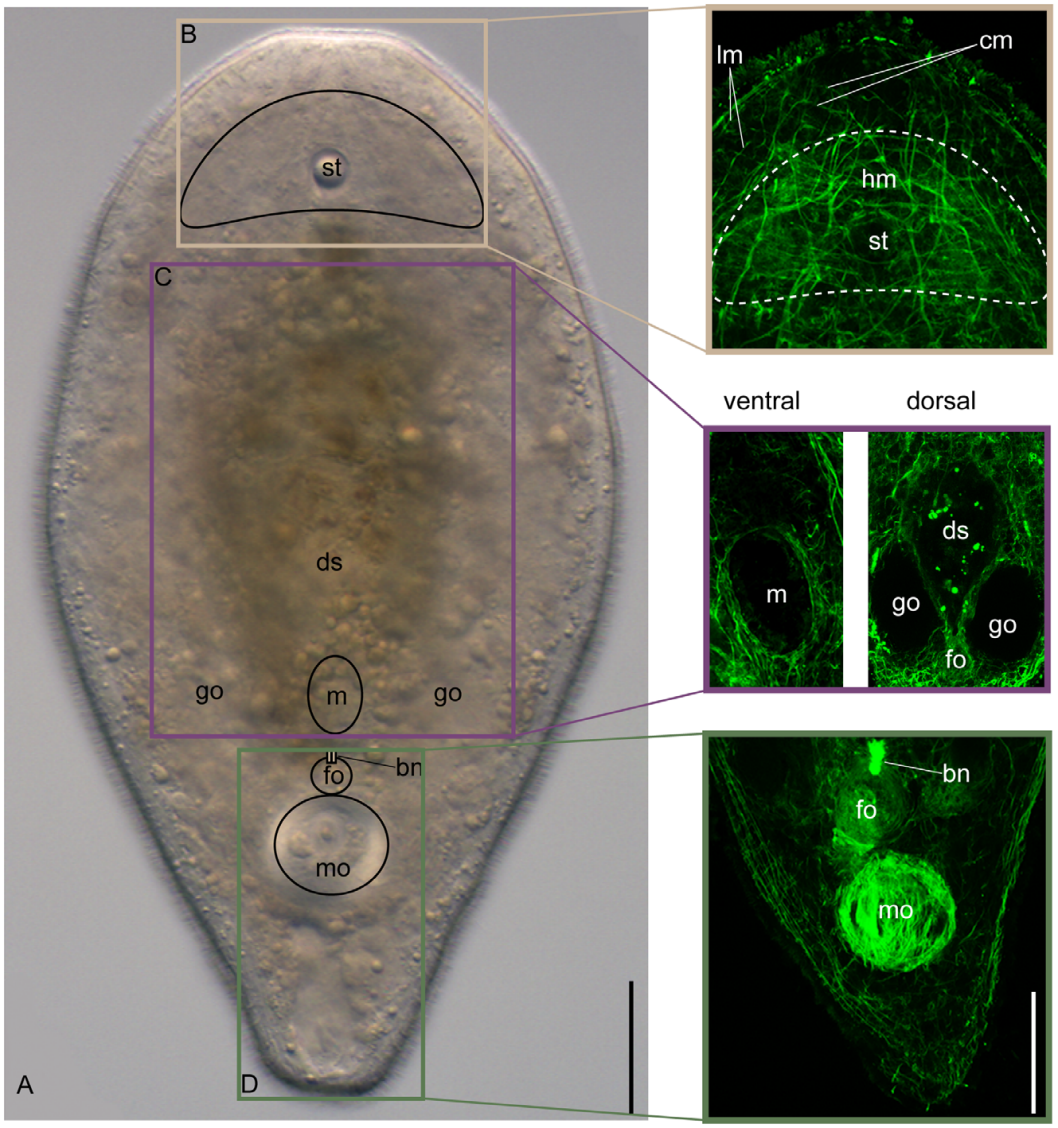
Anatomy of adult *Isodiametra pulchra.* Gross anatomy and confocal images of the musculature. **A.** Living adult under Differential Interference Contrast, showing the statocyst (st), digestive syncytium (ds) and gonads (go). Positions of following inconspicuous structures are outlined: mouth (m), bursal nozzle (bn), postero-ventral female organ (for) and male genital organ (mo). **B–D.** Musculature of anterior, central and posterior regions of body visualized by fluorescent phalloidin labeling. Note the dense net of parenchymal muscles in the anterior region (B dashed line). Scale bars are 50 µm in all aspects.

*I. pulchra* is an acoel that lives abundantly in the mud of the northeast Atlantic Ocean [30],[31]. The anterior end of the worm is easily recognized by the presence of a statocyst (Fig. 1A and B, st), which is surrounded by a dense net of ‘head myocytes’ (Fig. 1B, hm). These muscles are internal with respect to the body wall muscles, which consist of outer circular, inner longitudinal (Fig. 1B, cm and lm) and diagonal muscles [18], [25].

The mouth opens ventrally and it is surrounded by specialized ring muscles (Fig. 1C, ventral); a pair of thick parenchymal muscles, which are likely to be used for feeding and egg laying (see below), cross each other at a position dorsal to the mouth (Fig. 1C, dorsal).

The ventral female and male copulatory organs are strong muscular structures. The female genital organ (Fig. 1C, fo) is anteriorly delimited by a bursal nozzle (Fig. 1D, bn) and posteriorly by the male genital organ that includes a tubular penis (Fig. 1D, mo).

Gonads are paired, consisting of ventral ovaries and dorso-lateral testes [29]. The gonads are not lined by any tissue and lie in the parenchyma. The neoblasts, *i*.*e*., the acoel somatic stem cells, are also located in the parenchyma close to the gonads [28]. Hatchlings and juvenile worms of *I. pulchra* have a very similar body plan, although they lack the reproductive organs.

### Gene Selection and Orthology

All of the genes characterized in this study are orthologous to bilaterian mesoderm markers, and are also partly expressed in the endoderm of cnidarians (see Supporting Information Fig. S1 and Supporting Information References S1). Although some of the genes are not exclusively expressed in bilaterian mesoderm, they all play a broad role in bilaterian mesoderm patterning and therefore are justified as diagnostic markers. These genes are the orthologs of: *Mef2*, which can trigger either myogenesis or neurogenesis depending on splice variants in cnidarians and bilaterians [32], [33]; *Six1/2,* used in neurogenic and myogenic circuits in Cnidaria and Bilateria [34], [35], [36], [37]; *Pitx*, whose expression seems not to be germ layer specific in Bilateria, nevertheless it is consistently expressed in the coelomic mesoderm of the deuterostomes [38], [39], [40]; as it is the gene *Tbr*, whose expression varies from neural to endomesodermal in different taxa [41], [42], [43], [44], [45].

Finally, *FoxA* orthologs are central nodes of the endomesoderm gene regulatory network across the Bilateria [46]. Consistently, in the acoel *Convolutriloba longifissura*, *FoxA* is expressed in the endoderm during embryonic development and in freshly hatched worms [47]. However, *FoxA,* in *e.g* planarians and nematodes, is necessary to the proper development of the muscular pharynx [48], [49], [50].

The orthology assignments for all genes are given in the Supporting Information (Fig. S2, S3, S4, S5, S6, S7, S8, S9). In the case of the tropomyosin gene *IpTrp*, no phylogenetic analysis was conducted given the high amino-acid sequence similarity across all Eukaryota. *IpTrp* shares 90% of identical positions to a tropomyosin of another acoel species (*SrTrp*, Supporting Information Fig. S10) [51].

### Gene Expression

#### Genes that are broadly expressed in I. pulchra mesoderm (muscles, parenchyma, gonads and neoblasts): IpmuscleLIM, IpPitx IpFoxA1 and IpFoxC

**Figure 2:**
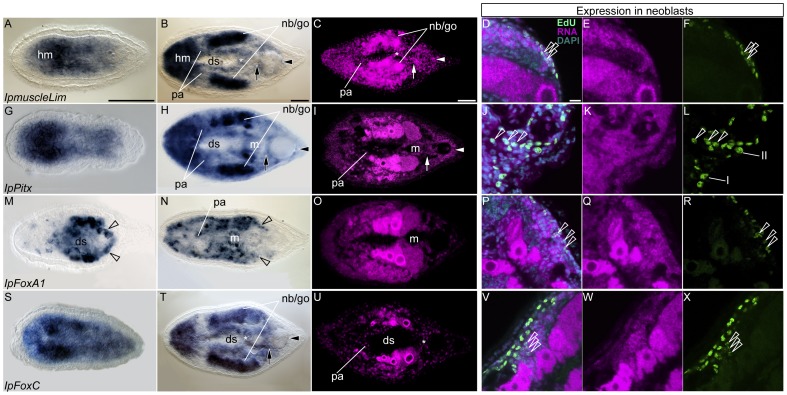
Expression of orthologs of bilaterian mesodermal genes that are broadly expressed in *I. Pulchra.* In the left panel whole-mount *in situ* hybridization of juvenile (left column) and adult (central and right columns) specimens are shown. Expression in the specimens in the right column is detected with fluorescent signal (purple). The arrow points to the female genital organ, arrowhead to the male genital organ. The asterisk indicates the cross parenchymal muscles. In the right panel the neoblasts localization of the transcript is shown. Left columns all show double stained worms with EdU and with the fluorescent antisense probe. The center and right columns show RNA transcripts and neoblasts staining alone. All aspects show a single confocal plane. The empty arrowheads point to colocalized EdU-RNA signal. Anterior to the left, scale bar 50 µm in all aspects**. A–F**. *IpmuscleLIM* expression. hm: head myocytes; nb/go: neoblasts and gonads pa:parenchymal cells; ds: digestive syncytium. **D–L**. *IpPitx* expression. The mouth is surrounded by specialized ring muscles (m) that express *IpPitx* (**H** and **I**). **M–R** *IpFoxA1* expression. *IpFoxA1* is expressed in the endodermal digestive syncytium (ds) as well as in the mesodermal musculature (*e.g.*, the mouth ring muscles, **N**). The black empty arrowheads in **M** and **N,** show paired muscles associated to the copulatory organs. **S–X**. *IpFoxC* expression.

*MuscleLIM* genes are expressed in muscles in a wide range of bilaterians [52], [53], [54] and cnidarians [55]. In *I. pulchra* juveniles *IpmuscleLIM* is expressed subepidermally along the whole anterior-posterior axis (Fig. 2A), with gradual decreasing expression from head, *i.e.*, the region of dense muscles net (Fig. 1B), to tail. In adult worms, the gene is strongly expressed in the anterior region and in two bilaterally symmetrical domains, whereas weaker expression has been observed in a cross domain between the digestive syncytium and the copulatory organs (Fig. 2B, asterisk).

The anterior domain includes the cell bodies of glands, epidermal cells, neurons and mostly myocytes [56] (Fig. 1B). The homogenous distribution of the *IpmuscleLIM* positive cells as well as the consistent muscle expression of *muscleLIM* orthologs across a wide range of metazoans, suggest that *IpmuscleLIM* is indeed expressed in the head myocytes. We showed that the two symmetrical *IpmuscleLIM* expression domains correlate with gonads and neoblasts by fluorescent *in situ* hybridization (FISH) (Fig. 2C), and by combined FISH-EdU labeling (Fig. 2D–F, open white arrowheads).

Finally, the cross domain of *IpmuscleLIM* expression corresponds to the cross muscles (compare Fig. 1C to Fig. 2C); the *IpMuscleLIM* positive cells surrounding the digestive syncytium in its anterior most part are parenchymal cells (Fig. 2B and 2C, pa).

IpPitx expression in juvenile worms mirrors IpmuscleLIM expression (Fig. 2G). Since in freshly hatched worms no peripheral parenchyma can be detected (Hejnol, Seaver and Martindale, unpublished data) [56], we feel confident in assigning IpPitx expression to the juvenile myocytes. Likewise, IpmuscleLIM and IpPitx are similarly expressed in adult worms (Fig. 2H). Clear muscular expression was detected in the genital organs and the mouth (Fig. 2I, arrow, arrowhead and m). Additionally, IpPitx is expressed in the parenchyma, gonads and in a subset of the neoblasts (Fig. 2I and J–L, open white arrowheads).

One of the two FoxA orthologs, IpFoxA1, is expressed in the juvenile digestive syncytium (Fig. 2M) whereas in adults its expression extends to the anterior mesoderm as well as to the peripheral parenchyma, the ring muscles encircling the mouth and to a pair of accessory muscles connected to the male copulatory organ (Fig. 2M and N, open arrowheads). By FISH, we detected expression in the gonads (Fig. 2O) as well as in the neoblasts, as confirmed by the co-localized EdU and FISH signals (Fig. 2 P–R).

The expression of *IpFoxC* is subepidermal and uniform along the antero-posterior axis of the juvenile (Fig. 2S). In adults, IpFoxC expression is restricted to specific domains similar to IpmuscleLIM and IpPitx (Fig. 2T). We detected anterior expression, likely in the “head-myocytes”, in the cross muscles and in the lateral domains encompassing both gonads and neoblasts (Fig. 2T, 2U and 2V–X).

All of the genes characterized in this section except *IpFoxA1* are expressed at the anterior animal pole of post-gastrulae embryos (S11 B, E and F), suggesting that they might be operating in a common (myogenic? [18]) gene regulatory network. *IpFoxA1* is instead expressed at the vegetal pole (S11 A) in the region that will form the endoderm.

The genes described in this section show a broad expression in I. pulchra juvenile and adult specimens and are expressed in muscles - or at least in a subset of them -, the peripheral parenchyma, in the gonads, and in a subset of neoblasts.

#### Mesodermal genes expressed in muscles, gonads and neoblasts of *I. pulchra*: *IpFoxA2, IpGATA456*, *IpMef2*, *IpSix1*/2

**Figure 3:**
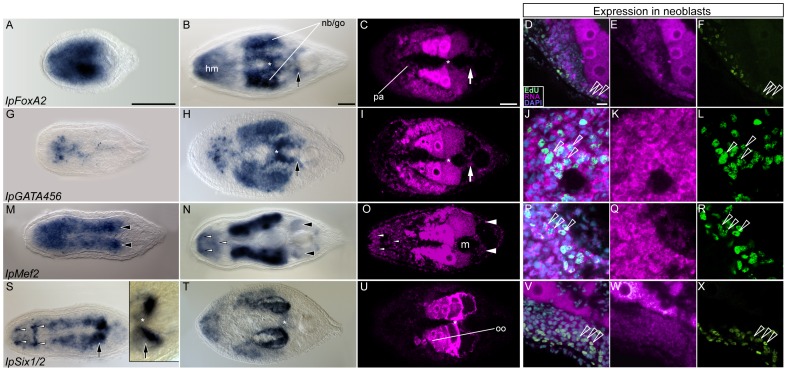
Orthologs of bilaterian mesodermal genes are expressed in subsets of endomesodermal tissues in *I. Pulchra*. The whole figure is structured as in Figure 2 with identical abbreviations and symbols, except when indicated. In the left panel, whole mount *in situ* hybridization are shown. The right panel shows the localization of the corresponding transcripts in the neoblasts, labeled by EdU. Anterior is to the left in all aspects and the scale bar is 50 µm**. A–F**. *IpFoxA2* expression. Signal in parenchymal cells (**C**) could only be detected by mean of fluorescent labeling. **G–L**. *IpGATA456* expression. **M–R**. *IpMef2* expression**.** The expression is stronger in the anlage of the copulatory organs (**M**, arrowheads). The small white arrowheads in **N** show likely neural expression of the gene in the anterior and posterior commissures of the brain. **S–X**. *IpSix1/2* expression. In (**S)** The small white arrowheads show neural expression of *IpSix1/2* in the juvenile. The inset is a magnification of the region of the copulatory organ, showing *IpSix1/2* expression in the anlage of the female copulatory organ (arrow) and cross parenchymal muscles (asterisk). oo: oocytes.

The second *FoxA* ortholog, *IpFoxA2*, is expressed subepidermally along the whole anterior-posterior axis of the hatchling, thus showing a broader expression domain than its paralog (Fig. 3A). The strongest expression of *IpFoxA2* is in the region of the digestive system, suggesting that the expression of the two *IpFoxA* paralogs overlaps in the digestive system during juvenile development (Fig. 3A). In adults, *IpFoxA2* expression is restricted to the head myocytes, the cross muscles (Fig. 3B) and to the gonads and neoblasts (Fig. 3C and 3D–F). The weak signal detected by FISH in the cells surrounding the digestive syncytium (Fig. 3C) is most likely background, given the lack of signal in the parenchyme in the more sensitive enzymatic reactions (Fig. 3B).

*IpGata456* is expressed anteriorly in scattered cells around and posterior to the statocyst of juveniles. The posterior *IpGATA456* positive cells are arranged along the midline (Fig. 3G). This expression domain persists in older worms. At this stage, *IpGata456* is additionally expressed in the cross muscles (Fig. 3H), in the gonads (Fig. 3I) and neoblasts (Fig. 3J–L).

*IpMef2* transcripts were detected in the head and in two longitudinal bands of cells in juveniles (Fig. 3M). *IpMef2* is highly expressed where the myocytes of the copulatory organs differentiate (Fig. 3M, arrowheads). In sexually mature worms, *IpMef2* is expressed in the anterior and posterior commissures of the brain (Fig. 3N, small white arrowheads), and weakly expressed between the two commissures, which we interpret as expression in the head myocytes (Fig. 3N). These results are consistent with neural and myogenic expression of *Mef2* orthologs as is typically seen in other eumetazoans [32],[33]. In adults, the gene is additionally expressed in close proximity to the male genital organ (Fig. 3N, arrowheads) as well as in the gonads (Fig. 3O) and in the neoblasts (Fig. 3P–R).

In juveniles, *IpSix1*/2 is expressed in the anterior and in two longitudinal bands of cells that flank the digestive syncytium (Fig. 3S). We infer that the anterior domain might correspond to neural expression, given that the strongest labeled spots coincide with the location of the two anterior and two posterior neurite loops of the brain, as well as in a transversal stripe which likely is the posterior brain commissure (Fig. 3S, small white arrowheads). The posterior connection of the two lateral expression domains of *IpSix1*/*2* corresponds to the developing female genital organ (Fig. 3S, inset). In adult worms *IpSix1/2* expression has considerably decreased with the exception of the gonads (Fig. 3T and U). Weak expression persists in the anterior region of cells that we infer to be myocytes based on distribution; even weaker expression is detected in the cross muscles (Fig. 3T and U, asterisk). By double EdU and FISH labeling, we detected *IpSix1/2* expression in neoblasts (Fig. 3V–X). In summary *IpFoxA2, IpGATA456*, *IpMef2*, *IpSix1*/*2* are expressed in myocytes, gonads and neoblasts, but are not expressed in cells of the peripheral parenchyma.

#### Genes expressed in a limited amount of cell types: *IpTwist1 and IpTwist2*, *IpTbr* and *IpTrp* (*tropomyosin*)

**Figure 4:**
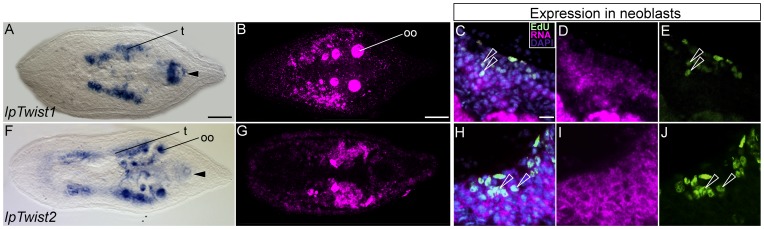
Expression of two *Twist* orthologs in adult specimens of *I. pulchra* . Left panel shows whole mount *in situ* hybridization and the right panels show expression of the two *Twist* orthologs in a subset of neoblasts. No expression was detected in juvenile specimens and therefore they are not shown. Anterior is to the left in all aspects. Scale bar is 50 µm. **A–B**. Expression of *IpTwist1* in adult specimens. Expression is detected in the male copulatory organ (arrowhead in A), in the testes (t) and oocytes (oo). **C–E**. Expression of *IpTwist1* in a subset of neoblasts. **F–J**. Expression of *IpTwist2* in adult specimens.

We have cloned two Twist orthologs (IpTwist1 and IpTwist2). Both orthologs did not show embryonic expression, while adult IpTwist1 expression is mainly restricted to the gonads and in the male copulatory organ (Fig. 4A and B). Double labeling with EdU revealed weak *IpTwist1* expression in a few neoblasts (Fig. 4 C–E). IpTwist2 expression overlaps with IpTwist1 in the gonads and in the copulatory organs (Fig. 4F) and several neoblasts are IpTwist2 positive as well (Fig. 4G and H–J). Although both Twist orthologs are expressed in all mesodermal cell types, namely myocytes, gonads and neoblasts, the expression domains are restricted to fewer cells than those of *IpFoxA2, IpGATA456*, *IpMef2*, *IpSix1*/*2*.

**Figure 5:**
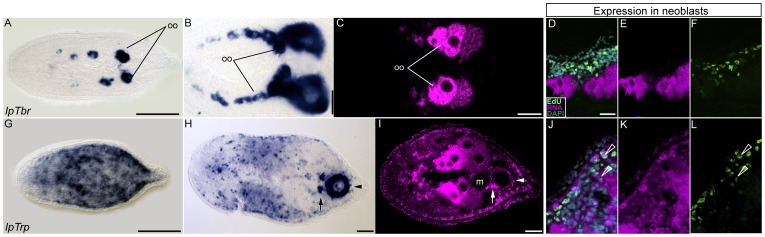
Expression of *IpTbr* and *IpTrp* (*tropomyosin*) in juvenile and adult *I. pulchra* specimens. Image as above, whole mount in situ hybridization in the left panel and colocalization of EdU and RNa signal in the right panel.Anterior is to the left and scale bar 50 µm in all whole-mount aspects. **A–C**. *IpTbr* expression, in one-week-old juvenile (A) and adult specimens (B and C). **D–F**. No expression of *IpTbr* was detected in any of the neoblasts. G-I. Expression of IpTrp in juvenile (G) and adult specimen (H and I). J–L. IpTrp is expressed in a subset of neoblasts that most likely already undertook the myocyte fate.

*IpTbr* is not expressed in hatchlings and only detected in late stage oocytes of juveniles (Fig. 5A). In mature adults *IpTbr* is expressed at all stages of oocyte development, i.e., from oogonia to mature oocyte (Fig. 5B–C). IpTbr seems only maternally expressed with a possible role in endomesodermal specification, indicated by its expression in the endomesoderm of embryos (Fig. S11 G). IpTbr transcripts did not colocalize with EdU labeling of neoblasts (Fig. 5D–F).

The general muscle marker IpTrp (tropomyosin) is broadly, but exclusively, expressed in the musculature of I. pulchra. Consistently, its expression is uniform in both juveniles and adults, but in the latter it is especially strong in the copulatory organs (Fig. 5H, arrow and arrowhead) which are the most muscular structure in adult I. pulchra and also in other acoels (Fig. 1D) [18], [51], [57], [58], [59].

Overall, we did not observe *IpTrp* expressed in neoblasts, although some IpTrp positive cells exhibited faint EdU labeling (Fig. 5J–L, see below), suggesting they could be neoblasts that have undergone differentiation (see discussion). To summarize, the two Twist orthologs characterized in this study are expressed in neoblasts, gonads and a subset of myocytes, but expression is restricted to few cells. Conversely, IpTbr and IpTrp are expressed in single cell type, the oocyte and the myocytes, respectively.

#### Considerations on gene expression in neoblasts

In the EdU assay, the fluorescent signal is detected by a modified uridine, which is incorporated in the nuclei of the proliferating cells. We observed two different patterns of incorporation into I. pulchra neoblasts. One type, called type I (after Gschwentner and colleagues, [60]) incorporate the uridine homogeneously at the periphery of the nucleolus (Fig. 2L). The others, type II neoblasts, incorporate the uridine in a less uniform fashion, so that the glowing nucleus has a granular aspect (Fig. 2L). We have observed that the genes IpFoxC, IpTwist1 and IpTwist2 are preferentially expressed in type II, granular neoblasts, whereas all other genes show no preference (Table 1). The genes IpmuscleLIM and IpPitx were expressed in all EdU-labeled neoblasts (data not shown) that we examined, whereas other investigated genes seemed to be expressed only in a subset of labeled neoblasts (Table 1). Finally the genes IpFoxA1, IpFoxC (Fig. 2P–R; Fig. 2V–X), IpFoxA2 (Fig. 3D–F) and IpTrp (Fig. 5J–L) were generally expressed in very few neoblasts, with a very low level of EdU incorporation (Table 1).

**Table 1:** Expression of mesodermal genes in *I. pulchra* neoblasts.

	Type II neoblasts	Low EdU signal	Type I and II neoblasts	Expressed in all examined neoblasts
*IpTwist1*	Yes			
*IpTwist2*	Yes			
*IpFoxC*	Yes	Yes		
*IpFoxA2*		Yes		
*IpFoxA1*		Yes		
*IpTrp*			Yes	
*IpGATA456*			Yes	
*IpMef2*			Yes	
*IpSix1/2*			Yes	
*IpmuscleLIM*			Yes	Yes
*IpPitx*			Yes	Yes

## Discussion

### Acoel Mesoderm and the Differential Expression of Mesodermal Genes in *I. pulchra* Musculature

Acoelomorphs have a unique early cleavage pattern (‘duet cleavage’) [26], [61], [62] and the fate map of the acoel species *Neochildia fusca* shows that the digestive system, the muscles and the peripheral parenchyma derive uniquely from the third pair of vegetal macromeres, the endomesodermal precursors [26].

Muscles in acoelomorphs are fibrous, mononucleated and of the smooth type. They are arranged in an orthogonal grid of inner-longitudinal and outer-circular muscles plus some diagonal muscles, interposed between the two other layers and crossing each other at the body midline [27]. In *I. pulchra*, specialized parenchymal muscles cross the body dorso-ventrally while specialized muscles are found around the mouth opening and the copulatory organs (Fig. 1D). Acoels do not possess body cavities; instead parenchymal tissue fills the space between the epidermis and the digestive syncytium. This tissue bears the parenchymal cells [27], [63], the neoblasts (*i.e.*, the acoel somatic stem cells) [28], [60] and the germ cells, plus all stages of gamete maturation (gonads). Gonads are not lined by any tissue (asacular) in any acoelomorph taxa [29], [64], [65]. The somata of epidermal, glandular and muscular cells are usually sunken below the body wall, making it difficult to distinguish them from the parenchymal cells. The anterior region of acoels is densely packed with myocytes, neurons, and scattered epidermal and gland cells, but neoblasts and parenchymal cells are usually absent from this area of the body [28], [56]. The posterior tip of the animal has no peripheral parenchyma but it is occupied by the myocytes of the copulatory organs, glands, and the spacious posterior chordoid vacuole (Fig. 1D).

**Figure 6:**
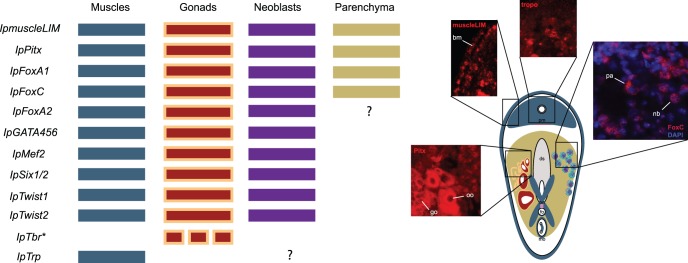
Summary of mesodermal gene expression in adult *I. pulchra*. Columns represent tissue types, rows the gene identity. Question marks represent detection ambiguities between standard and fluorescent *in-situ* hybridization protocols (see text for details). On the right side is a schematic representation of an adult worm. The tissue is color-coded according to gene expression on the left. Body wall and parenchymal muscles are in blue. Not all muscles are represented for clarity purposes. Peripheral parenchyma is in sandy-brown. Ovaries are in dark red and testes in light orange. Only single ovary and testes are represented not reflecting the real bilateral symmetric status of *I. pulchra* gonads. The same asymmetric representation is given for the neoblasts (dark purple). Examples of gene expression for each structure are given in the insets. bm: bodywall muscles; pm: parenchymal muscles; bn: bursal nozzle; fo: female copulatory organ; mc: male copulatory organ.

We show that all characterized genes are expressed in *I. pulchra* muscles, with the only exception being *Eomes/Tbrain/Tbx21* ortholog: *IpTbr* (Fig. 6).

Different muscles of *I. pulchra* express subsets of the genes (summarized in Table 2), whereas all express *IpTrp* (tropomyosin). Thus, in *I. pulchra*, different muscle types can be identified by specific gene expression profiles.

**Table 2:** Differential mesodermal gene expression in *I. pulchra* adult musculature.

Head muscles	Mouth ring muscles	Cross muscles	Female copulatory organ	Male copulatory organ
*IpTrp*	*IpTrp*	*IpTrp*	*IpTrp*	*IpTrp*
*IpmusleLIM*		*IpmusleLIM*	*IpmusleLIM*	*IpmuscleLIM*
*IpPitx*	*IpPitx*	*IpPitx*	*IpPitx*	*IpPitx*
*IpFoxA1*	*IpFoxA1*			
*IpFoxA2*		*IpFoxA2*	*IpFoxA2*	
*IpFoxC*		*IpFoxC*	*IpFoxC*	
*IpGATA456*		*IpGATA456*	*IpGATA456*	
*IpMef2*				*IpMef2*
*IpSix1/2*		*IpSix1/2*	*IpSix1/2*	
				*IpTwist1*
				*IpTwist2*

A substantial difference exists between male and female genital organ expression profiles (Table 2). The main difference between the two organs consists in the differential expression of genes that usually have important regulatory function during bilaterian myogenesis (*e.g., Mef2* and *Six1/2*) suggesting that the two organs are differentially regulated during development.

The female genital organ and the cross muscles express an identical set of genes and they have a common developmental origin, as exemplified by *IpSix1/2* expression in the anlage of the female copulatory organ during late juvenile development and its persistent expression in the adult cross muscles (Fig. 3S–U). The cross muscles, we think, might be used by the worm for laying the fertilized eggs through the mouth.

Indeed the silencing gene *IpPostHox*, which is expressed in a very similar expression domain than the ‘cross muscles’genes (listed in Table 2), produces worms incapable of ejecting the digested algae [66]. Likewise, we predict those worms would not be able to lay the eggs.

The myogenic specification factor IpMef2 is expressed in the anlagen of the copulatory organs and is not detected in the adult structure, which is consistent with a role in early myocyte specification as seen in other bilaterians [37]. In *Drosophila*, the gene *Twist* is an early myogenic factor that acts upstream of *Mef2* [67]. That seems not to be the case in *I. pulchra*, as both *IpTwist* orthologs are expressed in the developed copulatory organ. Because *Twist* can also behave as a myogeneic inhibitor, for example in the mouse [68], its function in the mature copulatory organs of *I. pulchra* is difficult to envision.

### Mesodermal Gene Expression in *I. pulchra*: Neoblasts, Gonads and Parenchyma

The neoblasts are the only dividing cells in the body of *I. pulchra* and they can differentiate into several cell types, presumably all, including germ cells [28]. It is generally believed that the metazoans germ cells evolved from totipotent somatic stem cells, similar to the acoelomorph neoblasts or the hydrozoan interstitial cells [69]. Key regulators of metazoan germ cell development (*e.g., piwi*) are also expressed in acoels and platyhelminth neoblasts as well as hydrozoan interstitial cells [28], [70], [71], [72]. In this study we extended the list of genes that are commonly expressed in the germ line and in the neoblasts of the acoel *I. pulchra*. With exception of the gene *IpTbr*, all mesodermal markers characterized here are expressed in the neoblasts and in the gonads of *I. pulchra* (Fig. 6). Remarkably, none of these orthologs are expressed in the stem cell system and/or in the germ line of platyhelminthes that have been investigated by large scale expression profiling [73], [74], [75], [76], [77].

The fact that all genes except *IpTbr* and, likely *IpTrp,* are expressed in neoblasts can indicate a mesodermal origin but it is likely that translation of these genes is temporary repressed by some mechanism (*e.g.,* RNA binding proteins [76], ) until activation of the differentiation pathway becomes necessary. The expression of the genes *IpFoxA1*, *IpFoxC* (Fig. 2P–R and 2V–X), *IpFoxA2* (Fig. 3D–F) and *IpTrp* (Fig. 5J–L) in cells with low EdU signal might also indicate that these cells already entered the post-mitotic phase (and therefore have reduced by half the uridine incorporation) and undertaken a differentiation pathway. We however consider this hypothesis less likely since the time frame between EdU incubation and fixation was very short.

Neoblasts and germ cells segregate during embryonic development, since they are already present in freshly hatched worms [28]. The expression of mesodermal genes in neoblasts and gonads, suggest that they arise from embryonic endomesoderm (Fig. S11).

The definition of the acoel parenchyma as ‘mesoderm’ is instead questionable.

**Figure 7:**
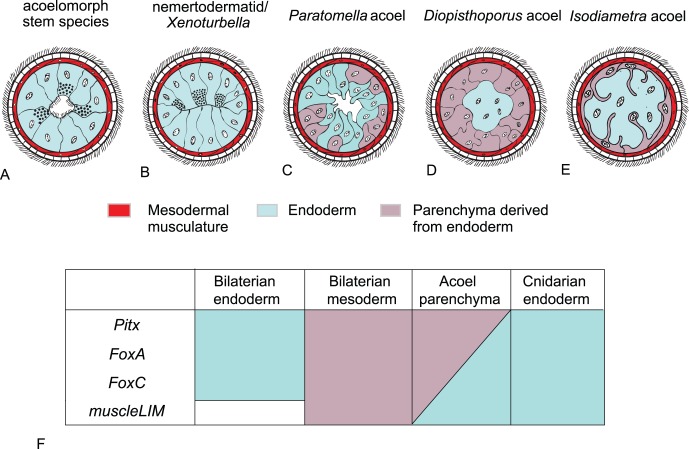
Tissue conditions in the digestive tract of different acoelomorph taxa. Schematic cross sections of the digestive tract of different acoel taxa (after Tyler & Smith [63]). **A.** Reconstructed ancestral condition of the acoelomorph stem species based on outgroup comparison (Cnidaria and/or Bilateria respectively). The epithelial digestive endoderm with lumen borders directly to the muscular grid. No parenchyma is present. **B.** Nemertodermatida and *Xenoturbella* posses an epithelial endoderm with gland cells, but lack a lumen. **C.** *Paratomella* (Acoela) possesses a digestive parenchyma in which no epithelial connections are present. Not all parenchymal cells are in contact with the digestive lumen. **D.** *Diopisthoporus* (Acoela) possesses a thick parenchymal layer that is forming a sheet around the digestive syncytium. **E.** Derived condition found in most acoel taxa including *Isodiametra*. Parenchymal cells surround the large syncytium but are only forming a relative thin sheet of cells with extensions into the digestive syncytium. **F.** Summary of gene expression in *I. pulchra* peripheral parenchyma and comparison of tissue specific orthologous gene expression in cnidarian and bilaterians.

In first instance the peripheral parenchyma only exists in the most derived acoel classes, those having a digestive syncytium whereas it is absent in acoelomorphs that have an epithelial digestive system [63], (Fig. 7 A–E). As such, it is unlikely that the peripheral parenchyma was part of the acoelomorph ground pattern, and it exemplifies how a tissue can evolve anew from the endoderm. Cell lineage experiments in the acoel *Neochildia fusca* have shown peripheral parenchyma differentiates from the same precursors of the mesodermal muscles and the endodermal digestive system (the endomesodermal precursors). However, it is not possible to see when the endodermal and mesodermal fates separate with these experiments.

With our gene expression study we have shown that the genes *IpmuscleLIM*, *IpPitx*, *IpFoxA1*, and *IpFoxC* are expressed in the acoel peripheral parenchyma. All these genes are of course expressed in the bilaterian mesoderm, but they all are also expressed in the cnidarian and bilaterian (except *muscleLIM*) endoderm [2], [32], [47], [79], [80], [81], [82], [83], [84], [85], [86] (Fig. 7F). Therefore, according to the position between the digestive syncytium and the ectoderm and without any homology statement, the peripheral parenchyma can be called “mesoderm” (see Ruppert [6]). However, to avoid an implied common evolutionary origin, we propose that it should be called ‘endodermal’ parenchyma, at least until more precise embryological data is available.

### Acoelomorphs as Derived Deuterostomes: does the Acoel Parenchyma Represent the Extant Vestige of an Ancestral Coelomic Cavity?

The phylogenetic position of the Acoelomorpha *sensu* Haszprunar [87], *i.e*., (Xenoturbellida+(Nemertodermatida+Acoela) is one of the most hotly debated topic in animal phylogeny, since two of the most recent phylogenomic studies propose two opposite positions for the clade. In one study that used massive taxon and gene sampling, the clade is placed as sister to all remaining bilaterians [21], while in the other study that uses a site-heterogenous model of protein evolution but much less molecular sequence data, the clade is placed within the deuterostomes [22]. The latter topology thereby implies the loss of several deuterostome diagnostic characters such as gill slits, enterocoely, and possibly a tripartite coelomic organization of the adult body plan [22].

**Figure 8:**
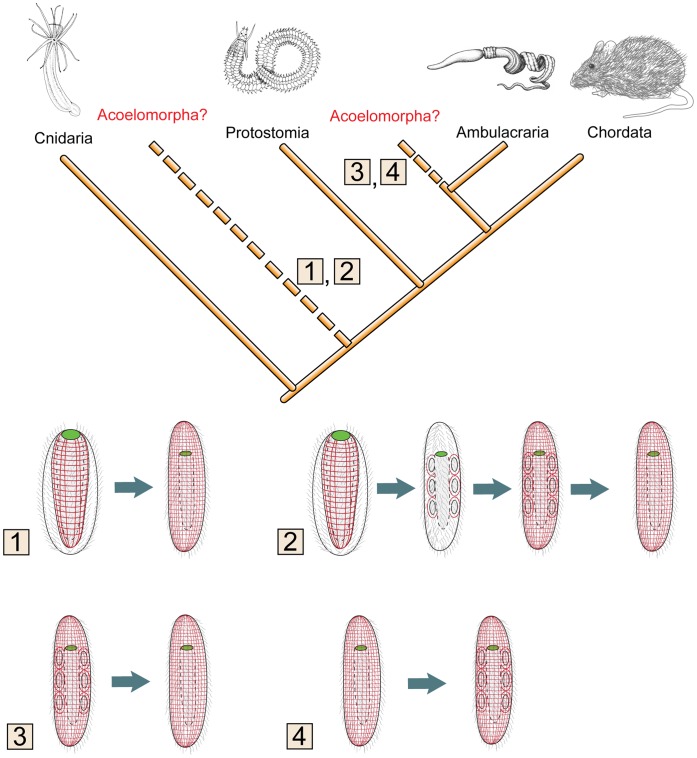
Different scenarios about mesoderm evolution depending on the phylogenetic position of Acoelomorpha. Two possible phylogenetic positions of Acoelomorpha either as sister to the remaining Bilateria (scenarios 1 and 2) or as sister group to Ambulacraria (scenarios 3 and 4). Musculature in red. Four possible scenarios are numbered. Scenario 1: A cnidarian-like ancestor with epithelial-muscle cells that form circular and longitudinal musculature form the orthogonal musculature of acoels. The musculature would be the first cell type of mesoderm [63]. Scenario 2: A similar cnidarian-like ancestor is forming myoepithelial coelomic cavities as outpouchings from the gastric cavity (according to enterocoely hypothesis [113]). In the lineage to the Acoelomorpha the orthogonal muscle grid of acoels is formed from the coeloms. After the formation of the muscle grid coeloms got reduced. This scenario includes several losses and gains and is thus not parsimonious and can be rejected. Scenario 3: In case the last common ancestor of Deuterostomia had coeloms, the coeloms got reduced in the lineage to the Acoelomorpha without any traces [12]. Scenario 4: Coelomic cavities of Ambulacraria are not homologous with those in other animal lineages [22] and are formed independently from the endoderm of a acoelomorph-like ancestor (*e.g.*, by enterocoely).

The origin of an acoelomate body plan from a coelomate ancestor is of course possible, given that it is observed in extant animal species, such as interstitial annelids [7], [8], [88] (Fig. 7). It is generally assumed that the acoelomate condition is achieved through progenesis [13], [88] and an attempt of deriving the acoelomorph body plan from neotenic juvenile hemichordates in which the coelom has not yet been formed, has been previously suggested [23]. The observation that in extant echinoderm species some body-wall muscles develop from the myo-epithelial coelomic lining where all progressive stages are present in a single specimen [89], has led some authors to generalize this model as the bilaterian model of muscle evolution [10], [15], [88]. However, there are no embryonic or adult traces of an anlage or degenerated coelom present in acoelomorphs. Thus, it remains unclear how the musculature might have separated from the former myo-epithelium of the coelomate ancestor (Fig. 8).

On first glance, an obvious conclusion would be that the acoel parenchyma represents the extant vestige of an ancestral coelom. There is some support in our gene expression data for this conclusion as, for example, the orthologs of *Pitx* and *FoxC* expressed in the acoel parenchyma (summarized in Fig. 6) are also expressed in the enterocoelic mesoderm of other deuterostomes [38], [39], [40] [80] [82]. However, this assumption leads to the unparsimonious implication that a peripheral parenchyma was present in the acoelomorph ancestor and must have been lost twice, in the lineages to xenoturbellids and the nemertodermatids (see discussion above and Fig. 7). We can exclude the enterocoelic formation of the peripheral parenchyma since it is nearly absent in hatchlings [56] (Hejnol, Seaver and Martindale, unpublished data); the endoderm is syncytial early in development and transient coelomic pouches are absent. The series of transitions from an epithelial digestive tract to the syncytial digestive system demonstrated by Smith and Tyler [63] (Fig. 7A–E) offers a more plausible explanation of parenchyma evolution, *i.e.,* as an acoel apomorphy (Fig. 7) that differentiates from neoblasts in late development. Therefore, even if acoelomorphs are deuterostomes, the parenchyma is unlikely to represent the remnant of a collapsed coelom.

### Acoelomorph as Derived Deuterostomes: are the Gonads the Vestige of the Ancestral Coelomic Cavity?

If the acoel peripheral parenchyma does not represent the extant vestige of a coelomic cavity, does the mesodermal gene expression in the acoel gonads support a coelomate acoelomorph ancestor? The assumption is plausible as the majority of bilaterian gonads are formed from coelomic cavities that are connected to the exterior through special ducts called gonocoels. Even though acoels do not have any of these structures, they still have genital openings, *i.e.,* the female and male genital organs, and even though the fertilized eggs are released through the mouth instead of the female genital organ, the acoel genital opening could be the reduced gonopores of an ancestral gonocoele. In this study we show that the genes *IpFoxC*, *IpGATA456*, *IpPitx*, *IpSix1/2* and *IpTbr* are expressed in neoblasts and/or gonads of *I. pulchra* (Fig. 6, Table 3), whereas the echinoderm orthologs are expressed in the coelomic mesoderm [39], [80], [90], [91], [92]. Likewise the *Branchiostoma* orthologs of *Mef2, Pitx*, *Six1*/*2*, *Tbr* and *Twist* - all expressed in *I. pulchra* neoblasts and/or gonads - are expressed in the Hatscheck’s diverticulum [38], [40], [44], [93], [94], [95] that forms by evagination from the anterior tip of the archenteron and is traditionally homologized to the protocoelic cavity of hemichordates [96] (but see Stach [97] for a different opinion). In addition, the lancelet’s orthologs of the genes *FoxC*, *Mef2*, *Six1*/2 and *Twist* are expressed in the larvae segmented mesoderm [82], [93], [94], [95], which develops through enterocoely [98]. Many of the genes for which we show expression in the gonads in *I. pulchra* (germ cells and differentiating gametes) are expressed in the coelomic lining of deuterostomes, making it plausible to recognize the acoel gonads as the remnant of the ancestral coelomic cavity of the deuterostomes (Table 3). Furthermore, because in those bilaterians having coelomic gonads, the germ cells develop (and evolved, see [69]) from somatic stem cells that are part of the germinative region of coelom epithelium [8], we might conclude that the neoblasts represent the remnant of the germinative region of the ancestral collapsed coelom. This hypothesis must be enriched by further data such as gene expression in other acoelomorph taxa. Especially relevant would be the investigation of orthologous gene expression in *Xenoturbella*, which has endodermal gonads, which under this scenario, may have failed to separate from the endodermal lineage. Accordingly we should expect to find the orthologous gonad-specific genes of *I. pulchra* (Fig. 6) to be expressed in the *Xenoturbella* endoderm.

**Table 3:** Acoel gonadal expression of mesodermal genes, compared to gene expression in the coelomic mesoderm of deuterostomes.

Acoel’s gonadal orthologs	Echinoderm coelomic mesoderm	Cephalochordate Hatscheck’s diverticulum	Early cephalochordate segmented mesoderm
*FoxC*	Yes	No	Yes
*GATA456*	Yes	Unknown	Unknown
*Mef2*	Unknown	Yes	Yes
*Pitx*	Yes	Yes	No
*Six1/2*	Yes	Yes	Yes
*Tbr*	Yes	Yes	No
*Twist*	No	Yes	Yes

Lastly, it must be noticed that the genes characterized in this study are not coelomic “markers”, but are more generally involved in mesoderm patterning across the Bilateria. Indeed, a *Tbr* ortholog is used to pattern the mesenchymal mesoderm in sea urchin, whereas it is expressed in the coelomic mesoderm of starfishes [92]. Thus co-option of the genes for patterning different tissues is common even among closely related species, and is even more likely to happen in more distantly related taxa such as acoelomorphs and echinoderms or cephalochordates.

To summarize, we cannot detect remnants of a former coelomic cavity in acoels. The coeloms of the coelomate ancestor must have disappeared without leaving any embryonic traces. This would be the first case of a complete coelomic reduction demonstrated in animals. Miniaturization per se does not necessarily imply that coeloms are lost (*e.g.,* interstitial priapulids [99] or hemichordates [100]). All clearly secondary acoelomate conditions show at least a coelom-anlage (interstitial polychaetes, [101], [102]) or the reduction of the coelom can be traced during embryogenesis as seen for example, in the anterior somitomeres of *Branchiostoma* [6] or in the dwarf male of the echiuran *Bonellia* [103]. Alternatively, the complete absence of the coelomic remnants could indicate the independent origin of the coelomic cavities in hemichordates and echinoderms, a possibility which has been suggested previously (Fig. 8) [8].

### Acoelomorphs as Sister Group to All Remaining Bilaterians: the Original State of Mesoderm and How New Mesodermal Tissues Evolved from the Endoderm

In contrast to the recently proposed deuterostomic affiliation of the Acoelomorpha, previous phylogenomic studies have placed the group as the sister to all remaining Bilateria [20], [21], thereby implying that some of the fundamental morphological and developmental traits of the group might be ancestral to the Bilateria. The cnidarians, which are the sister group to Bilateria, have ectoderm and endoderm as the only embryonic and adult tissue layers, although some polyps and most medusa stages have evolved individual muscle cells between the ectoderm and the endoderm [1], [2], [55], [104], [105]. In general, however, contractile cells of cnidarians are epithelio-muscular cells, *i.e.,* epithelial cells with basally concentrated contractile filaments [7], [8]. Possible scenarios of the evolution of myocytes involve either a detachment of the contractile basal portion from the apical-epithelial portion of the epithelio-muscular cell, or migration of single contractile cells into the space between endoderm and ectoderm [8], [14]. Given that cnidarian polyps have epithelio-muscular cells in both the ectoderm and the endoderm it is obvious that individual muscle cells can arise from both layers. It is indeed observed that the muscle cells of different metazoans can differentiate from the endoderm (*e*.*g*., ctenophores and acoels), ectoderm (*e*.*g*., hydrozoan jellyfishes), or both germ layers (*e*.*g*., spiralians and ecdysozoans) [26], [104], [105], [106], [107], [108].

One convincing answer to the question of whether the bilaterian mesoderm evolved from the endoderm or the ectoderm or from both tissue layers is offered by the expression of bilaterian mesoderm orthologs in anthozoan cnidarians [2], [32], [86], [109], [110], [111].

Most of the *Nematostella* (Anthozoa) orthologs of the genes characterized here are expressed in the endoderm, (although *FoxA, GATA* and *Mef2* orthologs are also expressed in the ectoderm [2], [32], [109]) as well as in the acoel musculature (Fig. 6, Table 2) [26]. In our opinion, this condition is most easily explained in a phylogenetic frame where the acoelomorphs are the sister group to the Nephrozoa, and consequently the muscles represent the most ancestral mesodermal cell type given that they were the unique mesodermal cells present in the acoelomorph ancestor (Fig. 8, scenario 1). Other mesodermal structures (*e.g.,* coeloms, and enterocoelic cavities) must have evolved later (Fig. 8). Thorough comparative molecular developmental investigations on protostome groups (*e*.*g*., Brachiopoda, and Chaetognatha) would further clarify if coeloms evolved once or multiple times in the Nephrozoa (see literature in Nielsen [96]).

The developmental origin of neoblasts in the Acoelomorpha is unclear, since fate mapping studies do not show a high enough resolution and need to be combined with early EdU labeling. At present, we can only predict that the neoblasts of nemertodermatids would express a similar set of genes to the acoels. Whether or not those neoblasts represent a subpopulation committed to endomesodermal fates whereas a second population segregate from the ectoderm and becomes committed to epidermal and neural differentiation, is an open question.

### Conclusions

In this study we show that most of the acoel homologs of bilaterian mesodermal transcription factors are also expressed in mesodermal compartments of the acoel, which only consist of muscles, gonads and neoblasts [26]. Our gene expression study suggests that some neoblasts and germ cells may have been derived from endomesodermal precursors and are thus true mesoderm. If the acoelomorphs are nested inside the deuterostomes [22], it is likely the acoelomate condition in acoels arose from a coelomate ancestor. However, we find no traces or anlage of mesodermal tissue that indicates the former presence of a coelom in a coelomate ancestor. Only the gonads could represent the ‘vestige’ of a secondary coelomic cavity. If the Acoelomorpha are the sister group to the remaining Bilateria [20], [21], mesoderm evolution by ‘enterocoely’ is less parsimonious. In this scenario, myocytes that form an orthogon of circular and longitudinal musculature are likely the first mesodermal cell type that evolved in Bilateria. Other mesodermal tissues such as coeloms or connective tissue must have evolved independently as secondary separations from the endoderm - similar to the secondary separation of the parenchyma in the acoel lineage. However, a solid phylogenetic framework of animals is needed to trace the path of mesoderm evolution and differentiation.

## Materials and Methods

### Gene Cloning and Orthology Assignment

Putative orthologs of genes of interest were identified by a BLAST search against *I. pulchra* transcriptome (Berezikov et al., manuscript in preparation) using known sequences. Gene orthology of *I. pulchra* sequences were tested by reciprocal blast against NCBI Genbank. For all the sequences supported by reasonable e-values, we designed pairs of gene specific primers or RACE primers, and we performed PCR on cDNA from *I. pulchra* juveniles, amplified with the SMARTer RACE cDNA Amplification kit (Clontech). PCRs were performed using the manufacturer instructions. Primer sequences are available on request. Amino acid alignments were made with MAFFT and corrected by hand for obvious alignment errors (NEXUS files are available upon request). MrBayes3.2 [112] was used to conduct a Bayesian phylogenetic analysis. The models used for each analysis were JTT+I+G. The results are a consensus of two converged runs of 2,000,000 (*fox* genes 50,000) generations sampled every 1000 generations and four chains. Gene accession numbers: *IpFoxA2:* JX853975, *IpFoxA1:* JX853976, *IpFoxC:* JX853977, *IpGata456:* JX853978, *IpmuscleLIM:* JX853979, *IpMef2:* JX853980, *IpPitx:* JX853981, *IpSix1*/*2*: JX853982, *IpTbr:* JX853983, *IpTrp:* JX853984, *IpTwist1:* JX853985, *IpTwist2:* JX853986.

### Animal Rearing and Labeling

Adult specimens of *Isodiametra pulchra* (Smith & Bush 1991) (formerly *Convoluta pulchra*) were reared as described by De Mulder et al. 2009 [28]. Ripe adults filled with oocytes were selected from culture plates and transferred to Petri dishes with filtered seawater and starved overnight. Deposited eggs were collected daily and fixed and processed for *in situ* labeling as described by Hejnol and Martindale [47]. To penetrate the eggshell, the fertilized eggs were treated with 0.01% Pronase (Sigma) and 0.1% thioglycolate (Sigma) in seawater, before fixation. Juveniles and adults were collected periodically and fixed for enzymatic *in situ* hybridization. Fluorescent *in situ* labeling was conducted using the TSA Plus Cy3 or Cy5 Kit (Perkin Elmer). Phalloidin stainings were conducted after a published protocol [59]. EdU-ClickIT labeling (Invitrogen) was performed following the manufacturer’s instructions after incubating starved worms for 2 h at room temperature in filtered artificial seawater containing EdU at a concentration of 100 µM.

### Documentation

Digital images of *in situ* hybridized specimens were taken with a microscope equipped with Nomarski optics and processed through Aperture 3.0 software (Apple inc.). Fluorescent-labeled specimens were analyzed with a SP5 confocal laser microscope (Leica, Germany) and processed by the ImageJ software 1.43 u (Wayne Rasband, NIH). Final figure plates and phylogenetic trees images were arranged with Photoshop CS3 and Illustrator CS3 (Adobe).

## Supporting Information

Figure S1 **Summary of gene expression in cnidarians and bilaterians.** Mesodermal expression is highlighted in red. Detailed references list is given below. The orthologs of tropomyosin are not included in the table given that the gene is expressed in muscular cells (among others) of all metazoans as it is in the Acoelomorpha. In the Cephalochordata, “Hatschek’s po” indicates the anterior pouch evaginating from the archenteron, *i.e.* coelomic mesoderm.
(TIF)

Figure S2 **Gene orthology assignment of** ***I. pulchra*** **forkhead genes.** Bayesian analysis of the orthology of the studied genes *IpFoxA2* (JX853975), *IpFoxA1* (JX853976), *IpFoxC* (JX853977).
(TIF)

Figure S3 **Gene orthology assignment of** ***I. pulchra*** **GATA genes.** Bayesian analysis of the orthology of the studied gene *IpGata456* (JX853978).
(TIF)

Figure S4 **Gene orthology assignment of** ***I. pulchra*** **muscle LIM gene.** Bayesian analysis of the orthology of the studied gene *IpmuscleLIM* (JX853979).
(TIF)

Figure S5 **Gene orthology assignment of** ***I. pulchra*** **Mef2 gene.** Bayesian analysis of the orthology of the studied gene *IpMef2* (JX853980).
(TIF)

Figure S6 **Gene orthology assignment of** ***I. pulchra*** **Six gene.** Bayesian analysis of the orthology of the studied gene *IpSix1*/*2* (JX853982).
(TIF)

Figure S7 **Gene orthology assignment of** ***I. pulchra*** **Pitx gene.** Bayesian analysis of the orthology of the studied gene *IpPitx* (JX853981).
(TIF)

Figure S8 **Gene orthology assignment of** ***I. pulchra*** **Tbr gene.** Bayesian analysis of the orthology of the studied gene *IpTbr* (JX853983).
(TIF)

Figure S9 **Gene orthology assignment of** ***I. pulchra*** **Twist genes.** Bayesian analysis of the orthology of the studied genes *IpTwist1* (JX853985) and *IpTwist2* (JX853986).
(TIF)

Figure S10 **Aminoacid sequence alignment of** ***I. pulchra*** **and** ***Symsagittifera roscoffensis*** **(Acoela) tropomyosin orthologs.**
(TIF)

Figure S11 **Embryonic expression of** ***I. pulchra mesodermal genes.*** All embryos shown are post-gastrulae embryos with the animal pole (asterisk) oriented upwards except in A. (*IpFoxA1*) and H. (*IpTrp*). Scale bar is 20 µm. **A.** Expression of *IpFoxA1* is at the vegetal pole (facing the reader) in gastrulating embryos. **B.** Expression of *IpFoxC* is expressed in putative mesodermal precursor cells along the animal vegetal axis (future anterior-posterior axis). **C.** *IpGATA456* is expressed in putative mesodermal blastomeres in the animal hemisphere only. **D.** *IpMef2* expression extends from the animal to the vegetal pole. **E.** *IpmuscleLim* follows the same pattern of expression of *IpMef2*. The embryo shown here is slightly younger than the embryo shown in D. **F.** *IpPitx* is expressed in the putative mesoderm of the animal hemisphere and its expression domain extends towards the vegetal posterior pole at later developmental stages (data not shown). **G.** *IpTbr* is expressed in all putative endomesodermal blastomeres along the animal-vegetal axis. **H.** *IpTrp* is expressed in differentiating myocytes. The animal-anterior pole, where the spiral muscle is formed [65] is marked by the asterisk.
(TIF)

References S1 **List of publications referenced in the Supporting Information.** The publications are cited in Supporting Information Figure S1 and Figure S11.
(DOC)
