# Supplementary figures and images for: Mesodermal Gene Expression in the Acoel Isodiametra pulchra Indicates a Low Number of Mesodermal Cell Types and the Endomesodermal Origin of the Gonads
Source: PLoS One. 2013 Feb 6;8(2):e55499. doi: 10.1371/journal.pone.0055499 (PMC3566195; doi:10.1371/journal.pone.0055499)

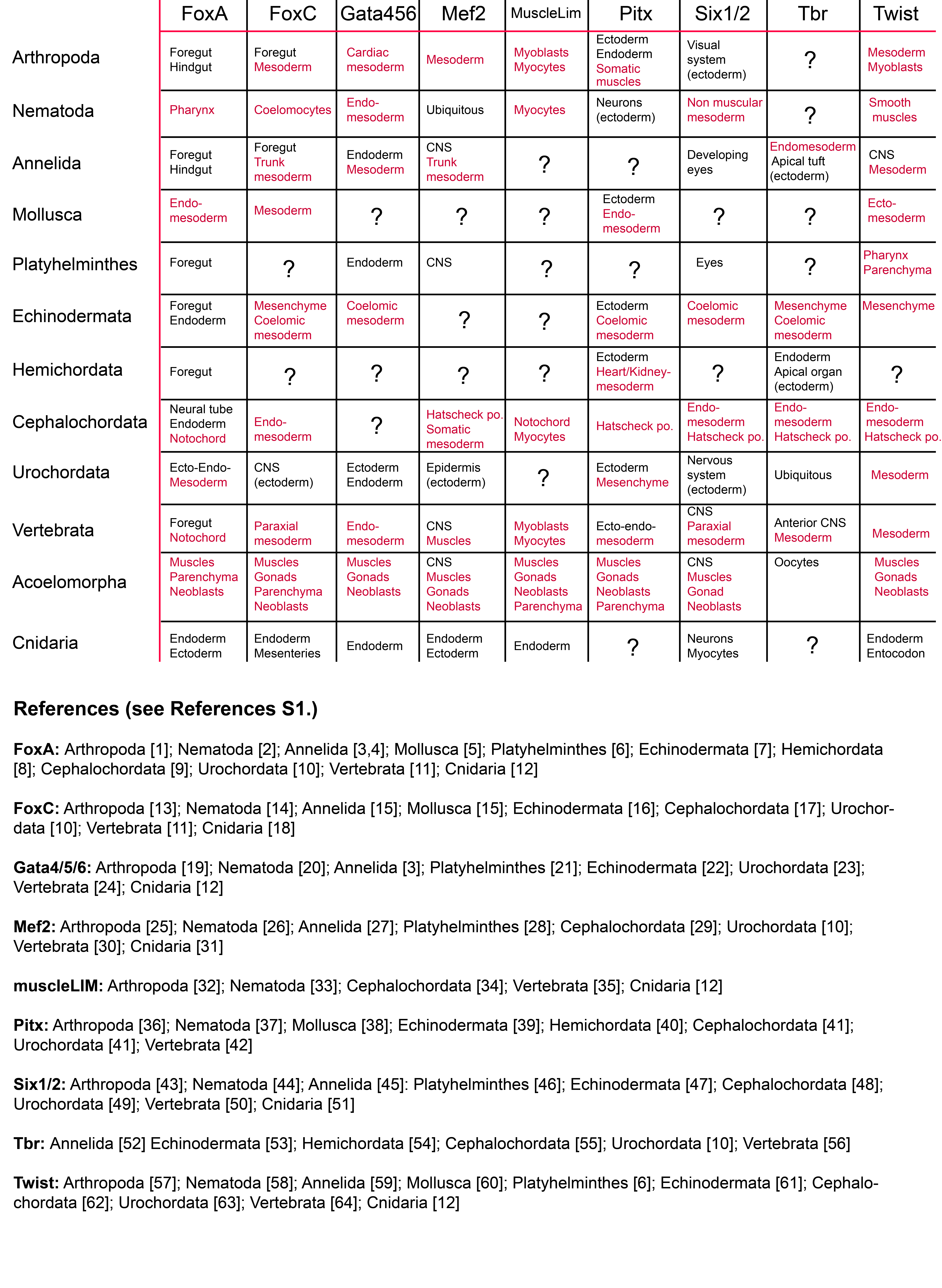

Supplement: Figure S1 — Summary of gene expression in cnidarians and bilaterians. Mesodermal expression is highlighted in red. Detailed references list is given below. The orthologs of tropomyosin are not included in the table given that the gene is expressed in muscular cells (among others) of all metazoans as it is in the Acoelomorpha. In the Cephalochordata, “Hatschek’s po” indicates the anterior pouch evaginating from the archenteron, i.e. coelomic mesoderm. (TIF) [file pone.0055499.s001.tif]

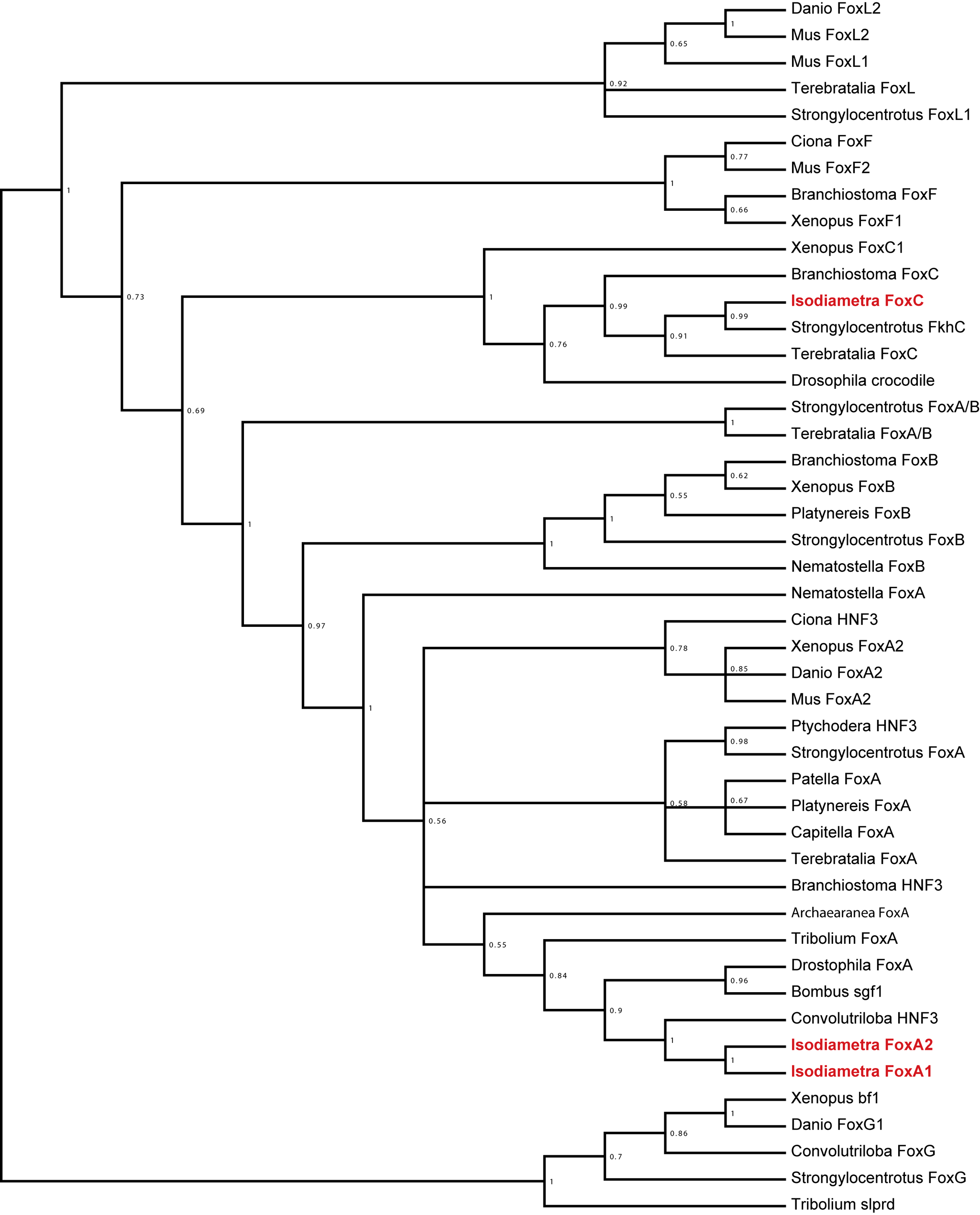

Supplement: Figure S2 — Gene orthology assignment of I. pulchra forkhead genes. Bayesian analysis of the orthology of the studied genes IpFoxA2 (JX853975), IpFoxA1 (JX853976), IpFoxC (JX853977). (TIF) [file pone.0055499.s002.tif]

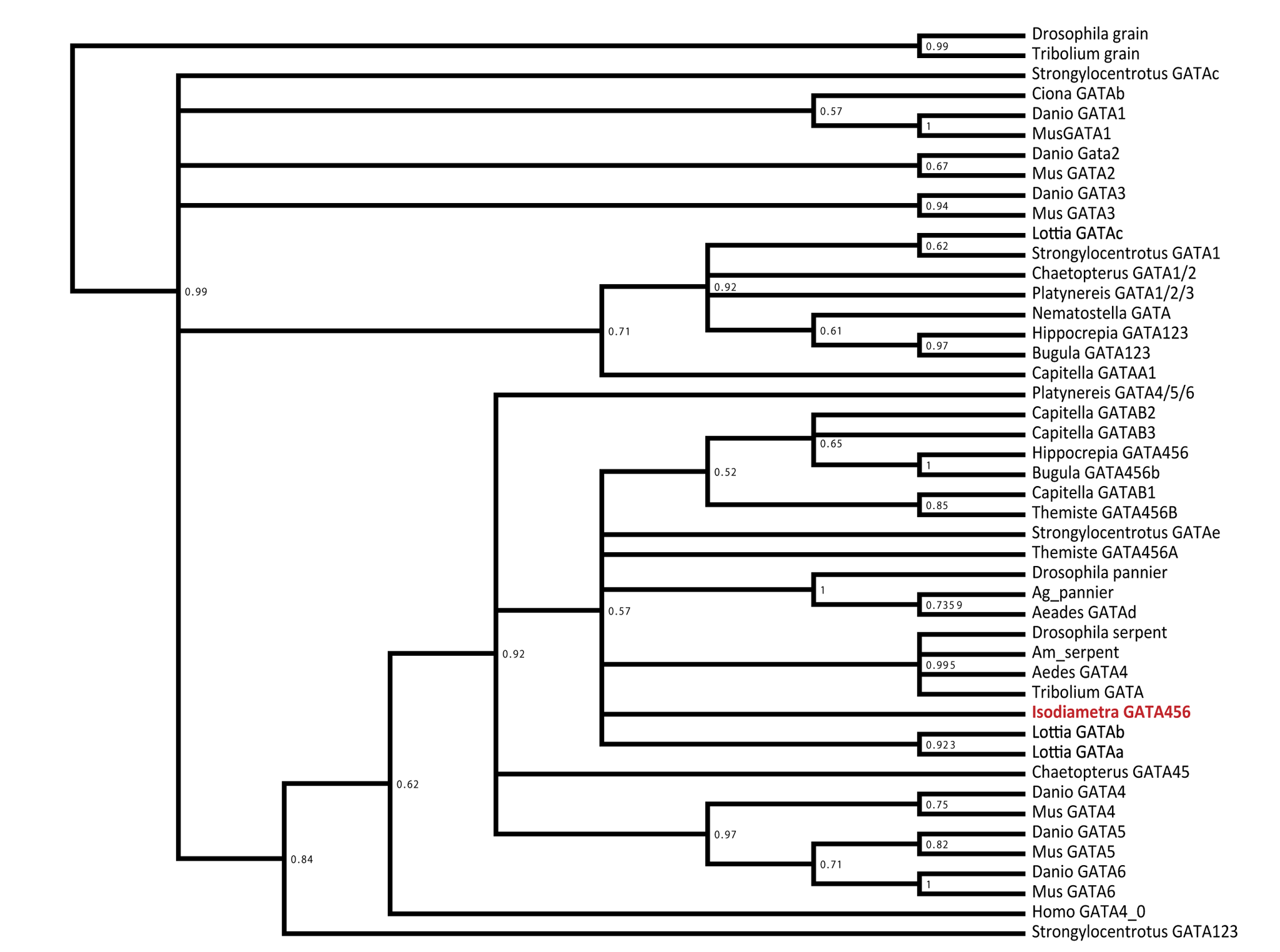

Supplement: Figure S3 — Gene orthology assignment of I. pulchra GATA genes. Bayesian analysis of the orthology of the studied gene IpGata456 (JX853978). (TIF) [file pone.0055499.s003.tif]

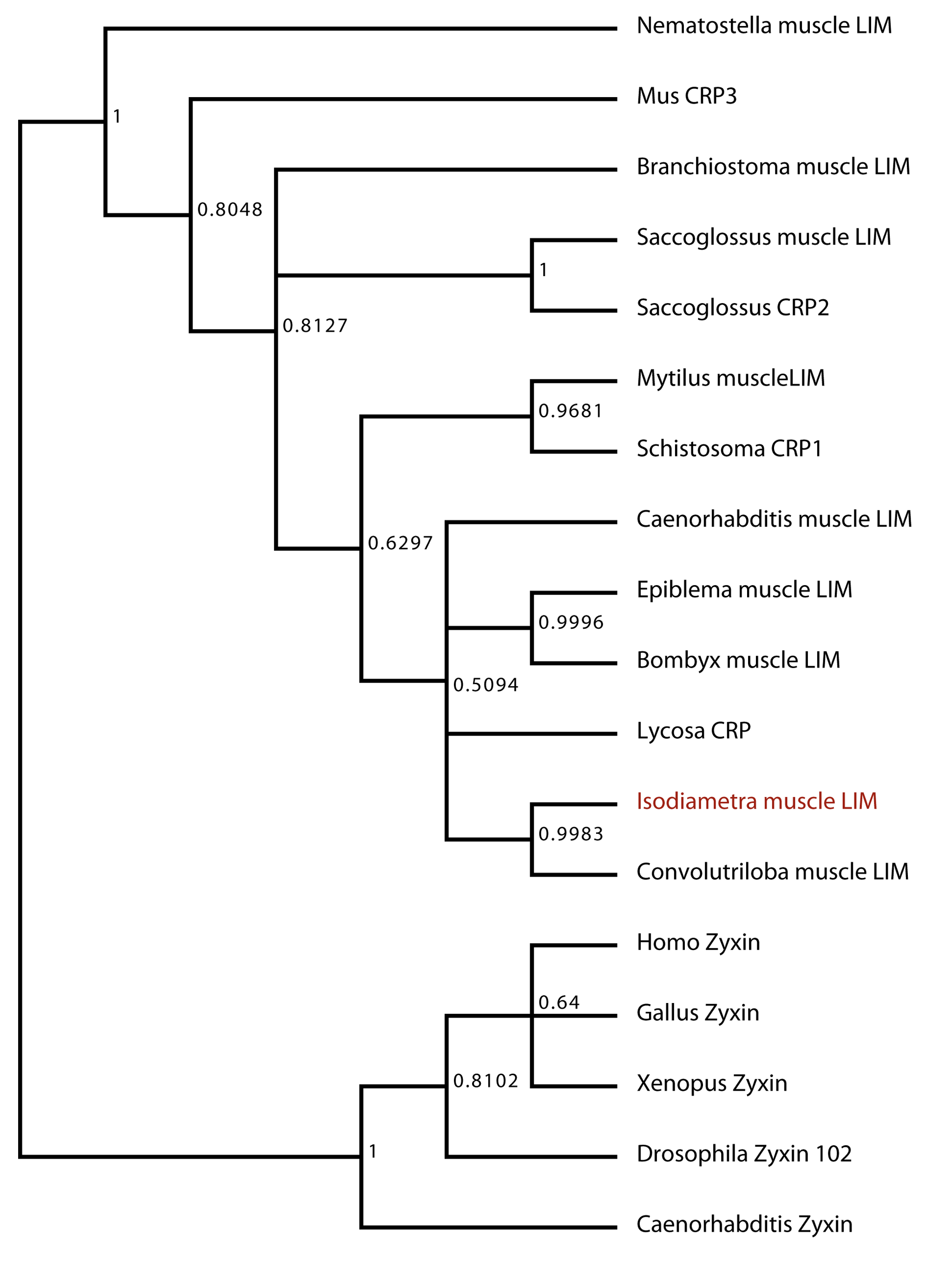

Supplement: Figure S4 — Gene orthology assignment of I. pulchra muscle LIM gene. Bayesian analysis of the orthology of the studied gene IpmuscleLIM (JX853979). (TIF) [file pone.0055499.s004.tif]

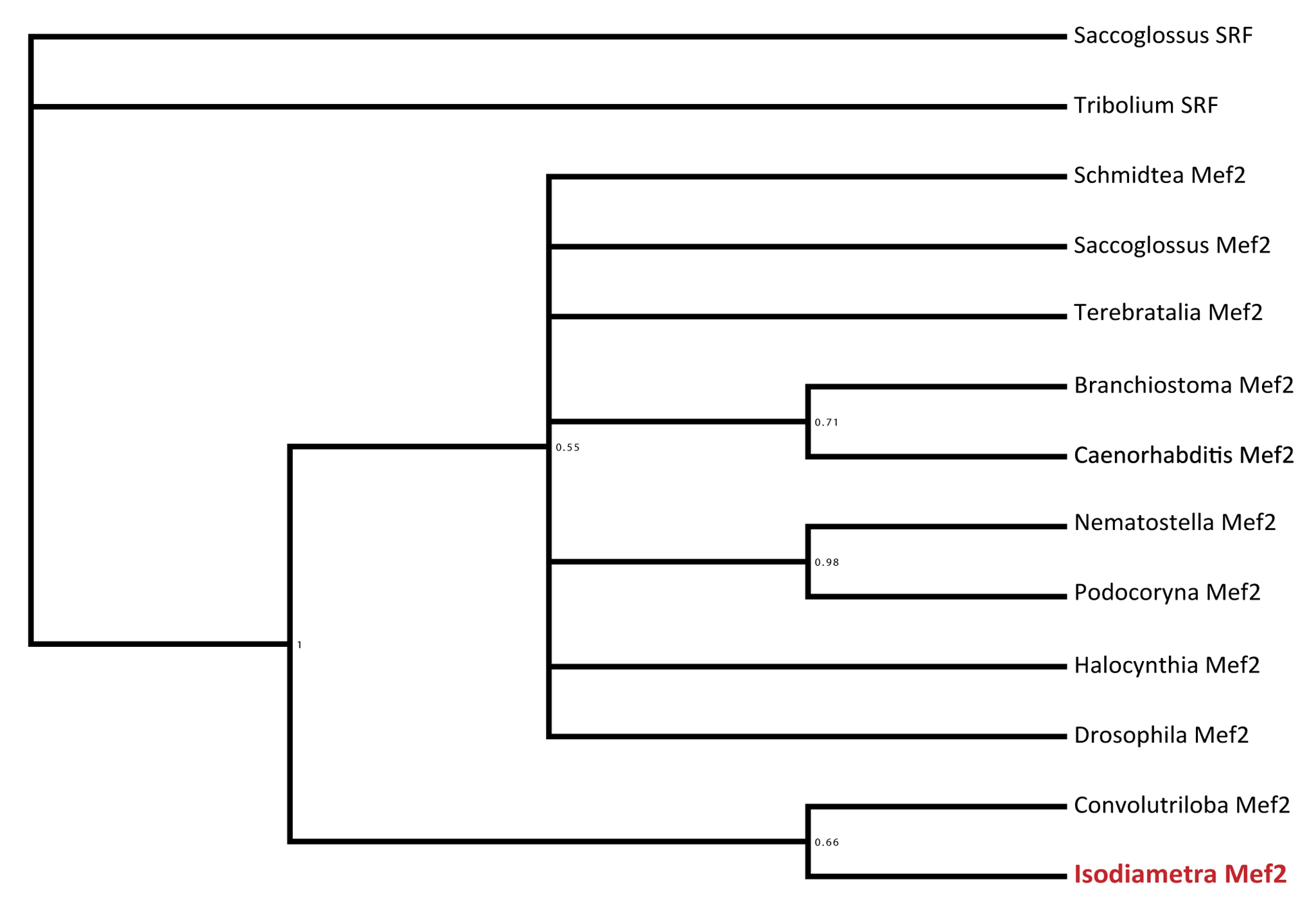

Supplement: Figure S5 — Gene orthology assignment of I. pulchra Mef2 gene. Bayesian analysis of the orthology of the studied gene IpMef2 (JX853980). (TIF) [file pone.0055499.s005.tif]

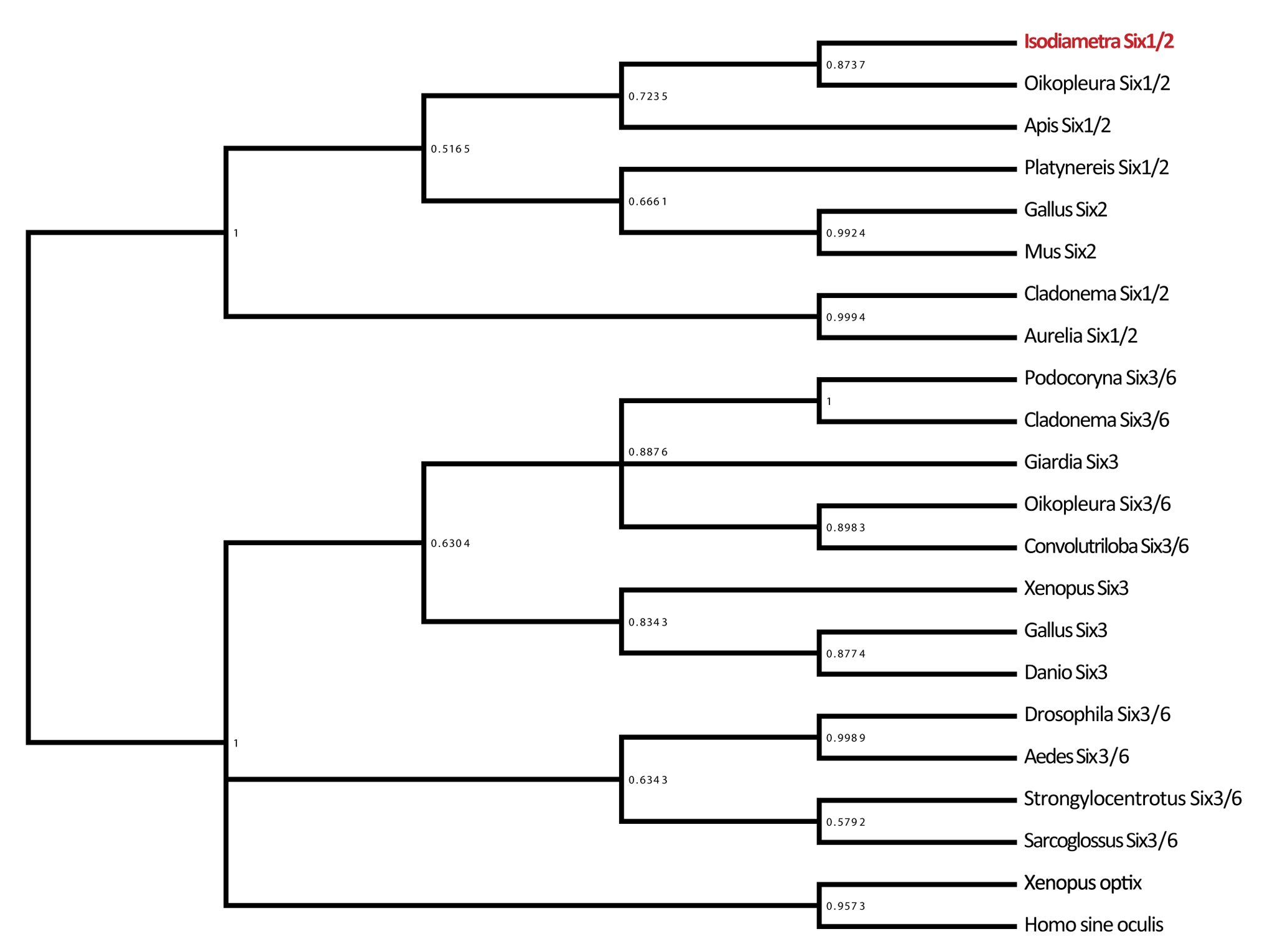

Supplement: Figure S6 — Gene orthology assignment of I. pulchra Six gene. Bayesian analysis of the orthology of the studied gene IpSix1/2 (JX853982). (TIF) [file pone.0055499.s006.tif]

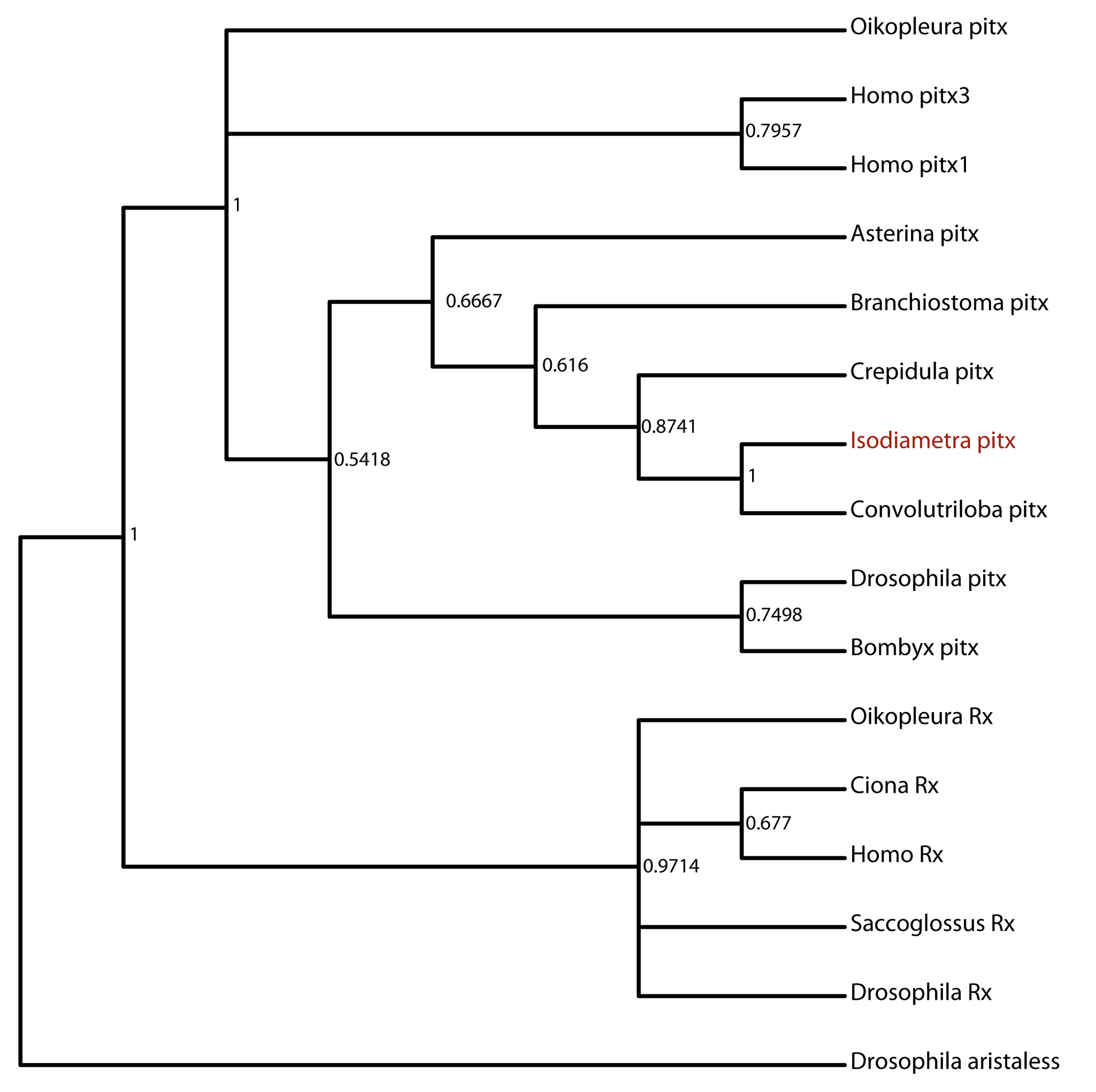

Supplement: Figure S7 — Gene orthology assignment of I. pulchra Pitx gene. Bayesian analysis of the orthology of the studied gene IpPitx (JX853981). (TIF) [file pone.0055499.s007.tif]

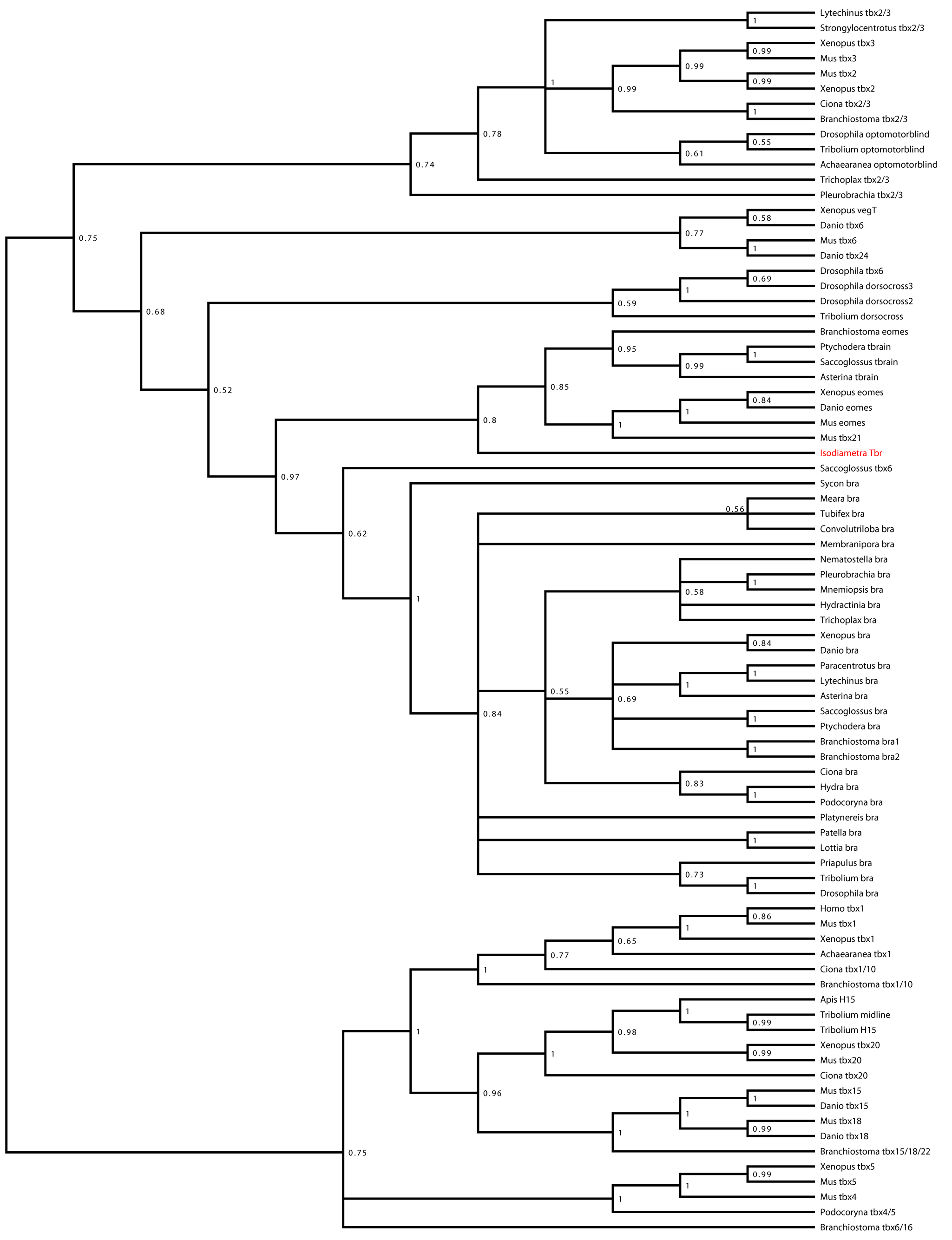

Supplement: Figure S8 — Gene orthology assignment of I. pulchra Tbr gene. Bayesian analysis of the orthology of the studied gene IpTbr (JX853983). (TIF) [file pone.0055499.s008.tif]

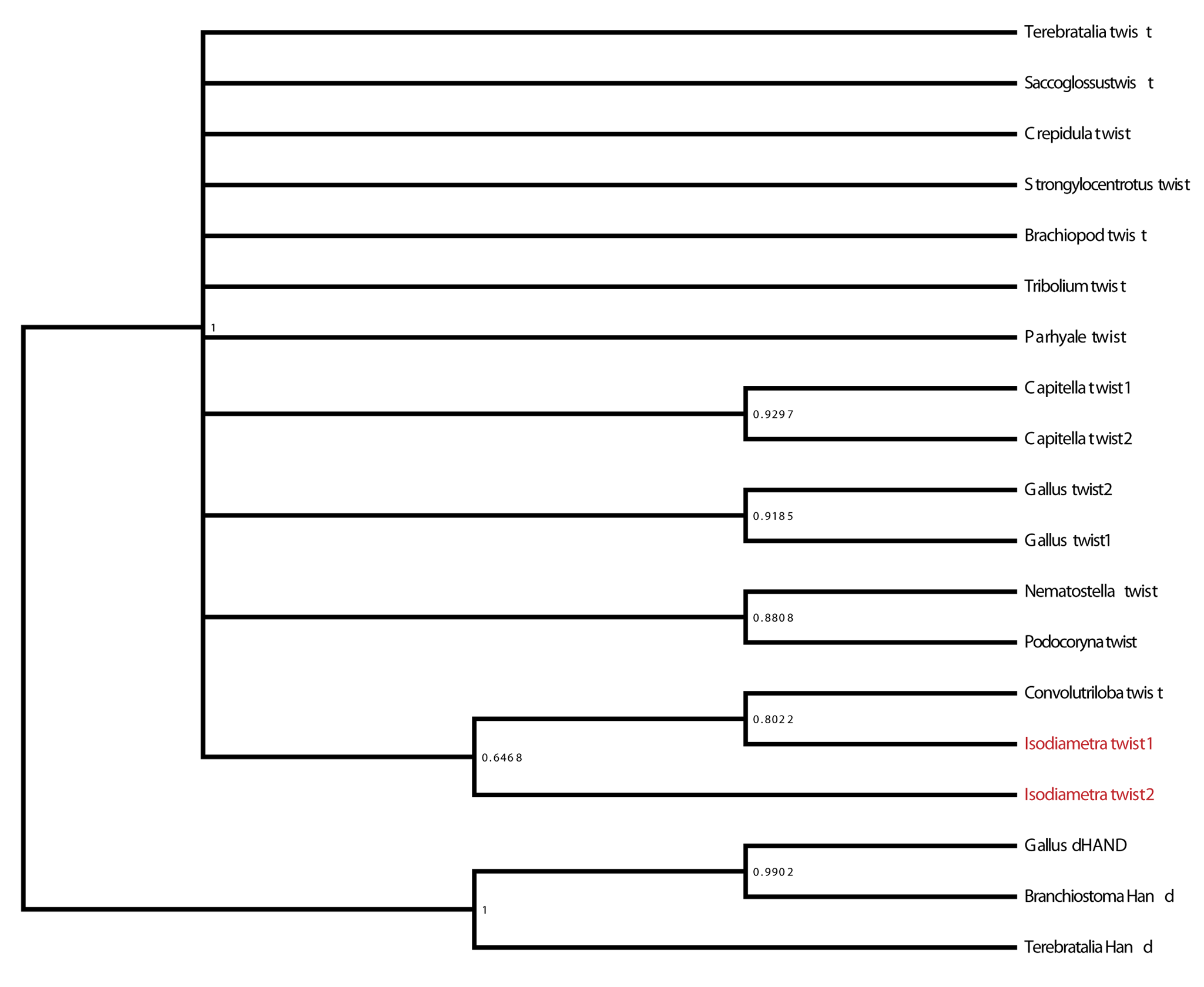

Supplement: Figure S9 — Gene orthology assignment of I. pulchra Twist genes. Bayesian analysis of the orthology of the studied genes IpTwist1 (JX853985) and IpTwist2 (JX853986). (TIF) [file pone.0055499.s009.tif]

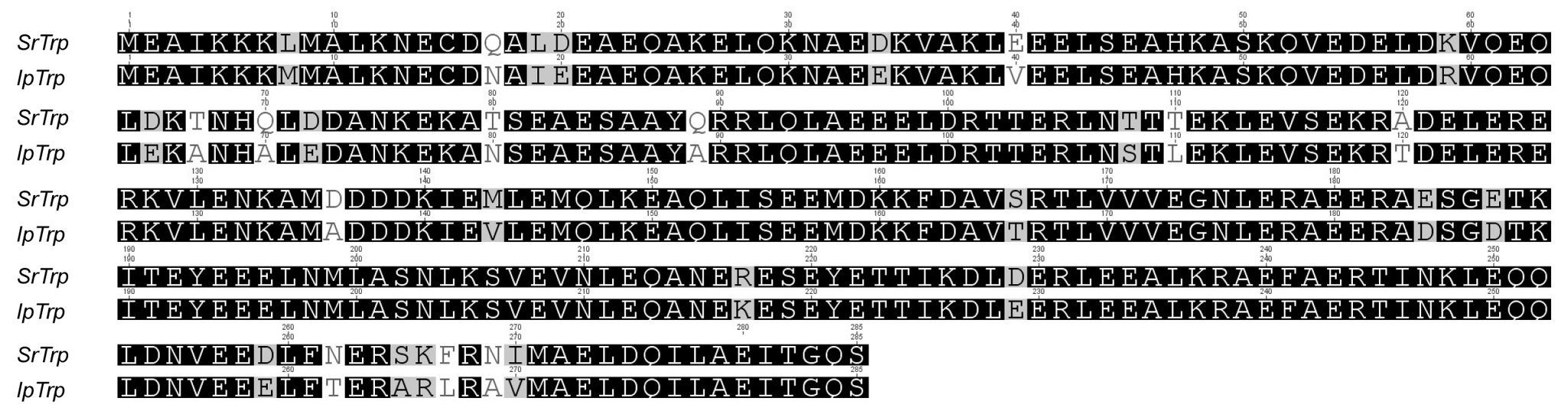

Supplement: Figure S10 — Aminoacid sequence alignment of I. pulchra and Symsagittifera roscoffensis (Acoela) tropomyosin orthologs. (TIF) [file pone.0055499.s010.tif]

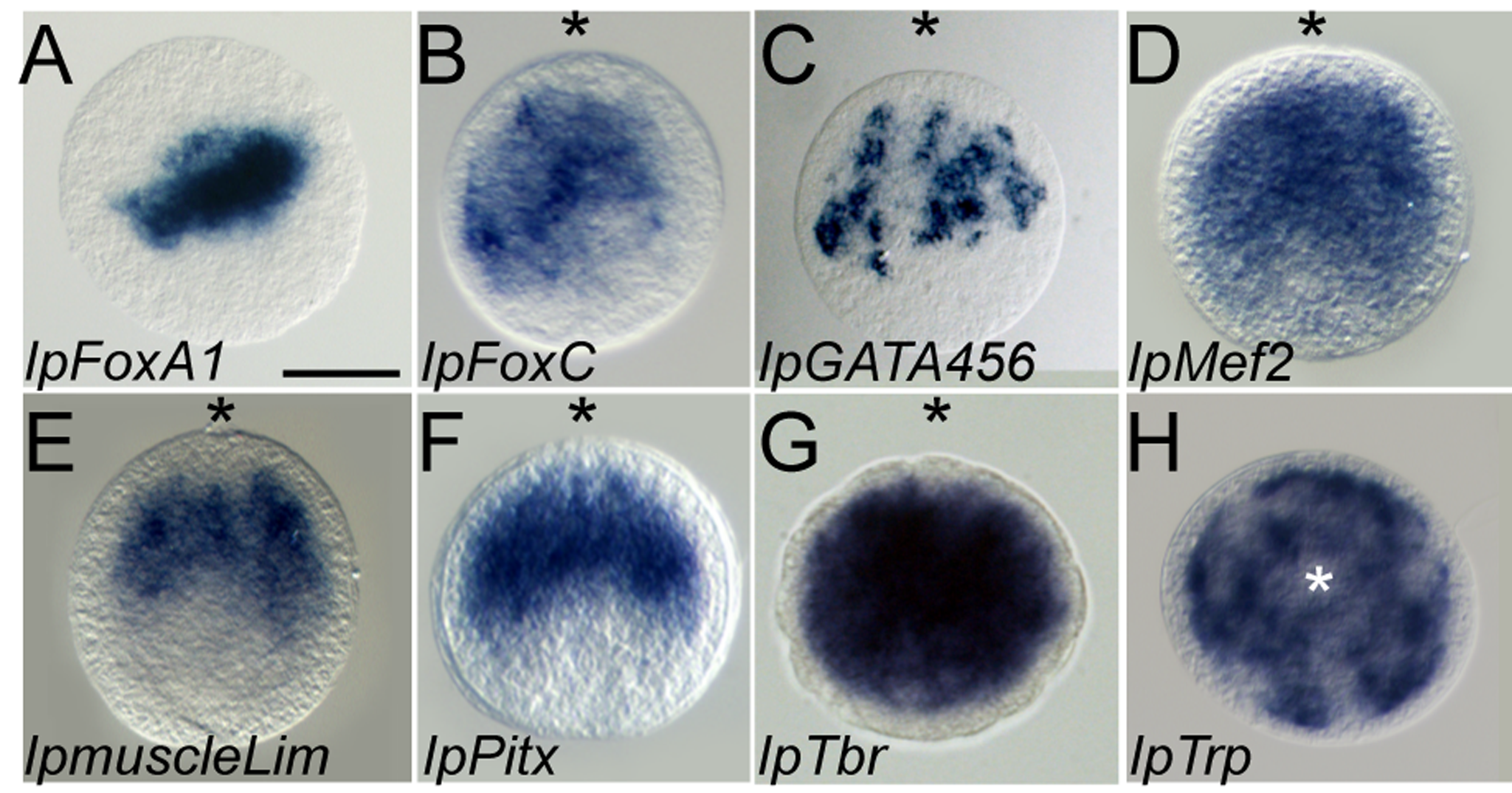

Supplement: Figure S11 — Embryonic expression of I. pulchra mesodermal genes. All embryos shown are post-gastrulae embryos with the animal pole (asterisk) oriented upwards except in A. (IpFoxA1) and H. (IpTrp). Scale bar is 20 µm. A. Expression of IpFoxA1 is at the vegetal pole (facing the reader) in gastrulating embryos. B. Expression of IpFoxC is expressed in putative mesodermal precursor cells along the animal vegetal axis (future anterior-posterior axis). C. IpGATA456 is expressed in putative mesodermal blastomeres in the animal hemisphere only. D. IpMef2 expression extends from the animal to the vegetal pole. E. IpmuscleLim follows the same pattern of expression of IpMef2. The embryo shown here is slightly younger than the embryo shown in D. F. IpPitx is expressed in the putative mesoderm of the animal hemisphere and its expression domain extends towards the vegetal posterior pole at later developmental stages (data not shown). G. IpTbr is expressed in all putative endomesodermal blastomeres along the animal-vegetal axis. H. IpTrp is expressed in differentiating myocytes. The animal-anterior pole, where the spiral muscle is formed [65] is marked by the asterisk. (TIF) [file pone.0055499.s011.tif]
