# Supplementary material for: Mesodermal Gene Expression in the Acoel Isodiametra pulchra Indicates a Low Number of Mesodermal Cell Types and the Endomesodermal Origin of the Gonads
Source: PLoS One. 2013 Feb 6;8(2):e55499. doi: 10.1371/journal.pone.0055499 (PMC3566195; doi:10.1371/journal.pone.0055499)
Supplement: References S1 — List of publications referenced in the Supporting Information. The publications are cited in Supporting Information Figure S1 and Figure S11. (DOC) [file pone.0055499.s012.doc]

**Supplementary References**

1. Schröder R, Eckert C, Wolff C, Tautz D (2000) Conserved and divergent aspects of terminal patterning in the beetle *Tribolium castaneum*. Proc Natl Acad Sci USA 97: 6591-6596.

2. Gaudet J, Mango SE (2002) Regulation of Organogenesis by the *Caenorhabditis elegans* FoxA Protein PHA-4. Science 295: 821-825.

3. Boyle M, Seaver E (2010) Expression of FoxA and GATA transcription factors correlates with regionalized gut development in two lophotrochozoan marine worms: *Chaetopterus* (Annelida) and *Themiste* lageniformis (Sipuncula). EvoDevo 1: 2.

4. Boyle MJ, Seaver EC (2008) Developmental expression of *foxA* and *gata* genes during gut formation in the polychaete annelid, *Capitella* sp. I. Evol Dev 10: 89-105.

5. Lartillot N, Le Gouar M, Adoutte A (2002) Expression patterns of *forkhead* and *goosecoid* homologues in the mollusc *Patella vulgata* supports the ancestry of the anterior mesendoderm across Bilateria. Dev Genes Evol 212: 551-561.

6. Martín-Durán JM, Amaya E, Romero R (2010) Germ layer specification and axial patterning in the embryonic development of the freshwater planarian *Schmidtea polychroa*. Dev Biol 340: 145-158.

7. de-Leon SB-T, Davidson EH (2010) Information processing at the foxa node of the sea urchin endomesoderm specification network. Proc Natl Acad Sci USA 107: 10103-10108.

8. Taguchi S, Tagawa K, Humphreys T, Nishino A, Satoh N, et al. (2000) Characterization of a hemichordate *forkhead/HNF-3* gene expression. Dev Genes Evol 210: 11-17.

9. Shimeld SM (1997) Characterisation of Amphioxus *HNF-3* Genes: Conserved Expression in the Notochord and Floor Plate. Dev Biol 183: 74-85.

10. Imai KS, Hino K, Yagi K, Satoh N, Satou Y (2004) Gene expression profiles of transcription factors and signaling molecules in the ascidian embryo: towards a comprehensive understanding of gene networks. Development 131: 4047-4058.

11. Carlsson P, Mahlapuu M (2002) Forkhead transcription factors: key players in development and metabolism. Dev Biol 250: 1-23.

12. Martindale MQ, Pang K, Finnerty JR (2004) Investigating the origins of triploblasty: `mesodermal' gene expression in a diploblastic animal, the sea anemone *Nematostella vectensis* (Phylum, Cnidaria; Class, Anthozoa). Development 131: 2463-2474.

13. Janssen R, Budd GE, Damen WGM (2011) Gene expression suggests conserved mechanisms patterning the heads of insects and myriapods. Dev Biol 357: 64-72.

14. Amin NM, Shi H, Liu J (2010) The FoxF/FoxC factor LET-381 directly regulates both cell fate specification and cell differentiation in *C. elegans* mesoderm development. Development 137: 1451-1460.

15. Shimeld SM, Boyle MJ, Brunet T, Luke GN, Seaver EC (2010) Clustered *Fox* genes in lophotrochozoans and the evolution of the bilaterian *Fox* gene cluster. Dev Biol 340: 234-248.

16. Tu Q, Brown CT, Davidson EH, Oliveri P (2006) Sea urchin Forkhead gene family: phylogeny and embryonic expression. Dev Biol 300: 49-62.

17. Mazet F, Amemiya CT, Shimeld SM (2006) An ancient *Fox* gene cluster in bilaterian animals. Curr Biol 16: R314-R316.

18. Magie C, Pang K, Martindale M (2005) Genomic inventory and expression of *Sox* and *Fox* genes in the cnidarian *Nematostella vectensis.* Dev Genes Evol 215: 618-630.

19. Fossett N, Hyman K, Gajewski K, Orkin SH, Schulz RA (2003) Combinatorial interactions of *Serpent*, *Lozenge*, and *U-shaped* regulate crystal cell lineage commitment during *Drosophila* hematopoiesis. Proc Natl Acad Sci USA 100: 11451-11456.

20. Coroian C, Broitman-Maduro G, Maduro MF (2006) Med-type GATA factors and the evolution of mesendoderm specification in nematodes. Dev Biol 289: 444-455.

21. Martín-Durán JM, Romero R (2011) Evolutionary implications of morphogenesis and molecular patterning of the blind gut in the planarian *Schmidtea polychroa*

Dev Biol 352: 164-176.

22. Lee PY, Nam J, Davidson EH (2007) Exclusive developmental functions of *gatae* cis-regulatory modules in the *Strongylocentrorus purpuratus* embryo. Dev Biol 307: 434-445.

23. Rothbächer U, Bertrand V, Lamy C, Lemaire P (2007) A combinatorial code of maternal GATA, Ets and β-catenin-TCF transcription factors specifies and patterns the early ascidian ectoderm. Development 134: 4023-4032.

24. Gillis W, St John J, Bowerman B, Schneider S (2009) Whole genome duplications and expansion of the vertebrate GATA transcription factor gene family. BMC Evol Biol 9: 207.

25. Ciglar L, Furlong EEM (2009) Conservation and divergence in developmental networks: a view from *Drosophila* myogenesis. Curr Opin Cell Biol 21: 754-760.

26. Dichoso D, Brodigan T, Chwoe KY, Lee JS, Llacer R, et al. (2000) The MADS-box factor CeMEF2 is not essential for *Caenorhabditis elegans* myogenesis and development. Dev Biol 223: 431-440.

27. Steinmetz P (2006) Comparative molecular end morphogenetic characterisation of larval body regions in the polychete annelid *Platynereis dumerilii.* Ph.D dissertation. Marburg: Philipps University Marburg.

28. Reddien PW, Bermange AL, Murfitt KJ, Jennings JR, S√°nchez Alvarado A (2005) Identification of genes needed for regeneration, stem cell function, and tissue homeostasis by systematic gene perturbation in planaria. Dev Cell 8: 635-649.

29. Zhang Y, Wang L, Shao M, Zhang H (2007) Characterization and developmental expression of *AmphiMef2* gene in amphioxus (abstract). Science in China Series C: Life Sciences 50: 637-641.

30. Potthoff MJ, Olson EN (2007) MEF2: a central regulator of diverse developmental programs. Development 134: 4131-4140.

31. Genikhovich G, Technau U (2011) Complex functions of *Mef2* splice variants in the differentiation of endoderm and of a neuronal cell type in a sea anemone. Development 138: 4911-4919.

32. Stronach BE, Renfranz PJ, Lilly B, Beckerle MC (1999) Muscle LIM proteins are associated with muscle sarcomeres and require dMEF2 for their expression during *Drosophila* myogenesis. Mol Biol Cell 10: 2329-2342.

33. Broday L, Kolotuev I, Didier C, Bhoumik A, Podbilewicz B, et al. (2004) The LIM domain protein UNC-95 is required for the assembly of muscle attachment structures and is regulated by the RING finger protein RNF-5 in *C. elegans*. J Cell Biol 165: 857-867.

34. Urano A, Suzuki MM, Zhang P, Satoh N, Satoh G (2003) Expression of muscle-related genes and two *MyoD* genes during amphioxus notochord development. Evol Dev 5: 447-458.

35. Arber S, Halder G, Caroni P (1994) Muscle LIM protein, a novel essential regulator of myogenesis, promotes myogenic differentiation. Cell 79: 221-231.

36. Vorbrüggen G, Constien R, Zilian O, Wimmer EA, Dowe G, et al. (1997) Embryonic expression and characterization of a *Ptx1* homolog in *Drosophila*. Mech Dev 68: 139-147.

37. Cinar H, Keles S, Jin Y (2005) Expression Profiling of GABAergic Motor Neurons in *Caenorhabditis elegans*. Curr Biol 15: 340-346.

38. Grande C, Patel NH (2009) Nodal signalling is involved in left-right asymmetry in snails. Nature 457: 1007-1011.

39. Duboc V, Röttinger E, Lapraz F, Besnardeau L, Lepage T (2005) Left-right asymmetry in the sea urchin embryo is regulated by nodal signaling on the right side. Dev Cell 9: 147-158.

40. Lowe CJ, Terasaki M, Wu M, Freeman RM, Jr., Runft L, et al. (2006) Dorsoventral patterning in hemichordates: insights into early chordate evolution. PLoS Biol 4: e291.

41. Boorman CJ, Shimeld SM (2002) *Pitx* homeobox genes in *Ciona* and amphioxus show left–right asymmetry is a conserved chordate character and define the ascidian adenohypophysis. Evol Dev 4: 354-365.

42. Gage PJ, Suh H, Camper SA (1999) The *bicoid*-related *Pitx* gene family in development. Mamm Genome 10: 197-200.

43. Cheyette BNR, Green PJ, Martin K, Garren H, Hartenstein V, et al. (1994) The *Drosophila sine oculis* locus encodes a homeodomain-containing protein required for the development of the entire visual system. Neuron 12: 977-996.

44. Amin NM, Lim S-E, Shi H, Chan TL, Liu J (2009) A conserved *Six-Eya* cassette acts downstream of Wnt signaling to direct non-myogenic versus myogenic fates in the *C. elegans* postembryonic mesoderm. Dev Biol 331: 350-360.

45. Arendt D, Tessmar K, de Campos-Baptista M-IM, Dorresteijn A, Wittbrodt J (2002) Development of pigment-cup eyes in the polychaete *Platynereis dumerilii* and evolutionary conservation of larval eyes in Bilateria. Development 129: 1143-1154.

46. Martín-Durán J, Monjo F, Romero R (2012) Morphological and molecular development of the eyes during embryogenesis of the freshwater planarian *Schmidtea polychroa*. Dev Genes Evol: 45-54.

47. Yankura K, Martik M, Jennings C, Hinman V (2010) Uncoupling of complex regulatory patterning during evolution of larval development in echinoderms. BMC Biol 8: 143.

48. Kozmik Z, Holland ND, Kreslova J, Oliveri D, Schubert M, et al. (2007) *Pax-Six-Eya-Dach* network during amphioxus development: conservation *in vitro* but context specificity *in vivo*. Dev Biol 306: 143-159.

49. Mazet F, Hutt JA, Milloz J, Millard J, Graham A, et al. (2005) Molecular evidence from *Ciona intestinalis* for the evolutionary origin of vertebrate sensory placodes. Dev Biol 282: 494-508.

50. Kawakami K, Sato S, Ozaki H, Ikeda K (2000) Six family genes—structure and function as transcription factors and their roles in development. BioEssays 22: 616-626.

51. Stierwald M, Yanze N, Bamert RP, Kammermeier L, Schmid V (2004) The *Sine oculis/Six* class family of homeobox genes in jellyfish with and without eyes: development and eye regeneration. Dev Biol 274: 70-81.

52. Arenas-Mena C (2008) The transcription factors *HeBlimp* and *HeT-brain* of an indirectly developing polychaete suggest ancestral endodermal, gastrulation, and sensory cell-type specification roles. J Exp Zool (Mol Dev Evol) 310B: 567-576.

53. Hinman VF, Nguyen AT, Cameron RA, Davidson EH (2003) Developmental gene regulatory network architecture across 500 million years of echinoderm evolution. Proc Natl Acad Sci USA 100: 13356-13361.

54. Tagawa K, Humphreys T, Satoh N (2000) *T-brain* expression in the apical organ of hemichordate tornaria larvae suggests its evolutionary link to the vertebrate forebrain. J Exp Zool 288: 23-31.

55. Satoh G, Takeuchi JK, Yasui K, Tagawa K, Saiga H, et al. (2002) *Amphi-Eomes/Tbr1*: an amphioxus cognate of vertebrate *Eomesodermin* and *T-Brain1* genes whose expression reveals evolutionarily distinct domain in amphioxus development. J Exp Zool B 294: 136-145.

56. Papaioannou VE (2001) T-box genes in development: From *Hydra* to humans. Int Rev Cytol: Academic Press. pp. 1-70.

57. Sandmann T, Girardot C, Brehme M, Tongprasit W, Stolc V, et al. (2007) A core transcriptional network for early mesoderm development in *Drosophila melanogaster*. Genes Dev 21: 436-449.

58. Harfe BD, Gomes AV, Kenyon C, Liu J, Krause M, et al. (1998) Analysis of a *Caenorhabditis elegans* *Twist* homolog identifies conserved and divergent aspects of mesodermal patterning. Genes Dev 12: 2623-2635.

59. Dill K, Thamm K, Seaver E (2007) Characterization of *twist* and *snail* gene expression during mesoderm and nervous system development in the polychaete annelid *Capitella* sp. I. Dev Genes Evol 217: 435-447.

60. Nederbragt AJ, Lespinet O, Van Wageningen S, Van Loon AE, Adoutte A, et al. (2002) A lophotrochozoan *twist* gene is expressed in the ectomesoderm of the gastropod mollusk *Patella vulgata*. Evol Dev 4: 334-343.

61. Wu S-Y, Yang Y-P, McClay DR (2008) *Twist* is an essential regulator of the skeletogenic gene regulatory network in the sea urchin embryo. Dev Biol 319: 406-415.

62. Yasui K, Zhang S-c, Uemura M, Aizawa S, Ueki T (1998) Expression of a *twist*-related gene, *Bbtwist*, during the development of a lancelet species and its relation to cephalochordate anterior structures. Dev Biol 195: 49-59.

63. Tokuoka M, Satoh N, Satou Y (2005) A bHLH transcription factor gene, *Twist-like1*, is essential for the formation of mesodermal tissues of *Ciona* juveniles. Dev Biol 288: 387-396.

64. Barnes RM, Firulli AB (2009) A twist of insight- the role of Twist-family bHLH factors in development. Int J Dev Biol 53: 909-924.

65. Ladurner P, Rieger R (2000) Embryonic Muscle Development of *Convoluta pulchra* (Turbellaria‚Acoelomorpha, Platyhelminthes). Dev Biol 222: 359-375.
